# Sequential Deposition and Remodeling of Cell Wall Polymers During Tomato Pollen Development

**DOI:** 10.3389/fpls.2021.703713

**Published:** 2021-07-27

**Authors:** Syeda Roop Fatima Jaffri, Cora A. MacAlister

**Affiliations:** Department of Molecular, Cellular and Developmental Biology, University of Michigan, Ann Arbor, MI, United States

**Keywords:** pollen, exine development, intine, cell wall, tomato, pectin, cellulose, callose (β-1, 3-glucan)

## Abstract

The cell wall of a mature pollen grain is a highly specialized, multilayered structure. The outer, sporopollenin-based exine provides protection and support to the pollen grain, while the inner intine, composed primarily of cellulose, is important for pollen germination. The formation of the mature pollen grain wall takes place within the anther with contributions of cell wall material from both the developing pollen grain as well as the surrounding cells of the tapetum. The process of wall development is complex; multiple cell wall polymers are deposited, some transiently, in a controlled sequence of events. Tomato (*Solanum lycopersicum*) is an important agricultural crop, which requires successful fertilization for fruit production as do many other members of the Solanaceae family. Despite the importance of pollen development for tomato, little is known about the detailed pollen gain wall developmental process. Here, we describe the structure of the tomato pollen wall and establish a developmental timeline of its formation. Mature tomato pollen is released from the anther in a dehydrated state and is tricolpate, with three long apertures without overlaying exine from which the pollen tube may emerge. Using histology and immunostaining, we determined the order in which key cell wall polymers were deposited with respect to overall pollen and anther development. Pollen development began in young flower buds when the premeiotic microspore mother cells (MMCs) began losing their cellulose primary cell wall. Following meiosis, the still conjoined microspores progressed to the tetrad stage characterized by a temporary, thick callose wall. Breakdown of the callose wall released the individual early microspores. Exine deposition began with the secretion of the sporopollenin foot layer. At the late microspore stage, exine deposition was completed and the tapetum degenerated. The pollen underwent mitosis to produce bicellular pollen; at which point, intine formation began, continuing through to pollen maturation. The entire cell wall development process was also punctuated by dynamic changes in pectin composition, particularly changes in methyl-esterified and de-methyl-esterified homogalacturonan.

## Introduction

The production of pollen was a major innovation during the evolution of land plants. The pollen grain is a highly specialized structure, which includes a complex and unique cell wall supporting its functions. The mature pollen wall can be broadly divided into two layers, an outer sporopollenin-rich exine and an inner intine, which has a composition similar to other plant cell walls. In addition to the differences in their composition, these two layers have markedly different developmental origins and functions (Heslop-Harrison, 1968). The exine protects the pollen cytoplasm from excess dehydration and other environmental insults and mediates adhesion to pollinators and the stigmatic surface (Zinkl et al., 1999). Once the relevant interactions between pollen and stigma take place, the pollen grain hydrates and the intine serves as the organizing site for the formation of a pollen tube. Once initiated, the pollen tube expands at its tip to complete pollen germination. The pollen tube will then carry the sperm nuclei from the pollen grain through the female floral tissue to a receptive ovule (Taylor and Hepler, 1997).

Pollen development takes place in the anthers, beginning in early immature buds, with the specification of microspore mother cells (MMCs) and proceeding through meiosis, microspore mitosis, and pollen development to mature pollen release at anther dehiscence (Canales et al., 2002; Scott et al., 2004; Gómez et al., 2015). During this process, the cell wall is radically altered with many cell wall polymers deposited, removed, or remodeled. The mature pollen cell wall contains material produced by both the developing pollen itself and by the sporophytic tapetum, a layer of cells lining the locule cavity in which the microspores develop (Owen and Makaroff, 1995).

Before the initiation of meiosis, the MMCs are surrounded by a simple primary cell wall; during early prophase, the middle lamella expands, followed by a reduction in the flanking fibrillous wall layers and the initiation of callose (β-1,3-glucan) deposition between the plasma membrane and the primary wall (Polowick and Sawhney, 1992). The deposition of callose continues during and after meiosis, eventually producing a thick callose wall, which holds the four products of meiosis in a tetrad (Polowick and Sawhney, 1993a; Owen and Makaroff, 1995). The callose wall serves as a scaffold for exine development, first by the deposition of primexine, a poorly defined microfibrillar polysaccharide matrix formed by the microspore (Hess and Frosch, 1994). Secretion of callase by the tapetal tissue causes the breakdown of the callose wall separating the individual microspores (Chasan, 1992; Polowick and Sawhney, 1993b; Rhee et al., 2003; Quilichini et al., 2014). The primexine serves as a scaffold for the deposition of sporopollenin produced by the tapetum. The sporopollenin polymerizes onto the primexine, producing the foot layer (Quilichini et al., 2014). The exine may be further elaborated with additional sporopollenin deposition, e.g., production of inner-column-shaped baculae and the surface decoration tectum. Together, the sporopollenin foot layer (nexine), tectum, and baculae (sexine) form the mature pollen exine (Polowick and Sawhney, 1992; Chebli et al., 2012; Jiang et al., 2013; Renzaglia et al., 2020). After exine deposition, the tapetal cell layer breaks down, releasing material for the outermost layer of the pollen, the pollen coat also called “pollenkitt” or “tryphine” (El-Ghazaly and Jensen, 1986). The pollen coat is required for the pollen-stigma interface and promotes pollen hydration (Ishiguro et al., 2010), and is largely composed of proteins and lipids (Piffanelli and Murphy, 1998; Bashir et al., 2013; Rejón et al., 2016).

Concurrent with the later stages of exine formation, the intine develops. Unlike the exine, which is composed primarily of specialized sporopollenin, the composition of intine is similar to that of other plant cell types with major components, including cellulose, pectin, and cell wall-associated proteins (Hess, 1993; Suárez-Cervera et al., 2002; Persson et al., 2007; Fang et al., 2008). The apertures, which will serve as possible sites for pollen tube initiation and pollen germination, develop a unique pollen wall, completely or partially lacking exine and, in some species, highly elaborating the intine into a specialized structure called the “Zwischenkörper” (Picken, 1984; El-Ghazaly and Jensen, 1986; Dobritsa and Reeder, 2017).

Mutants with defects in pollen wall formation have been identified in several species, providing insight into the molecular basis of pollen wall development and the roles of cell wall components. The process of exine deposition is relatively well studied, and there are many well-characterized exine mutants with male sterility phenotypes (Blackmore et al., 2007; Dobritsa et al., 2011; Jiang et al., 2013; Shi et al., 2015a). Broadly speaking, these mutants can be classified into overlapping functional categories, including biosynthesis and transport of sporopollenin precursors, primexine formation, and tryphine synthesis. Proteins falling in the sporopollenin biosynthesis class include ACYL-CoA SYNTHETASE5 (ACOS5), the fatty acyl reductase MALE STERILE2 (MS2), along with its rice ortholog DEFECTIVE POLLEN WALL (DPW), and acyl-CoA-binding proteins (AtACBP4, 5, and 6), among others (Aarts et al., 1997; Souza et al., 2009; Shi et al., 2011; Zhang et al., 2011; Hsiao et al., 2015). Since the synthesis of sporopollenin precursors takes place in the tapetum, they must be transported into the anther locule, which requires the ATP-binding cassette transporter superfamily member ABCG26/WBC27 (Quilichini et al., 2010; Choi et al., 2011). Notable primexine genes include Arabidopsis *DEFECTIVE IN EXINE FORMATION 1* (*DEX1*) and its rice ortholog *OsDEX1*(Yu et al., 2016). DEX1 is a membrane calcium-binding protein, and *dex1* pollen has reduced primexine and abnormal exine (Paxson-sowders et al., 2001). Examples of tryphine synthesis mutants include mutants of phosphoserine phosphatase, an enzyme required to catalyze the last step of the phosphorylated pathway of serine biosynthesis (PPSB). These mutants show a normal exine without any tryphine (Flores-Tornero et al., 2015).

Cellulose synthesis and callose synthesis and degradation are also required for pollen wall development. Arabidopsis triple mutants in the cellulose synthase complex members *cesa6, cesa9*, and *cesa2* produced deformed pollen with abnormally thickened intines, possibly due to a compensation by other cell wall polymers in the absence of cellulose (Persson et al., 2007). In Arabidopsis, the callose synthase CalS5 is required for callose wall formation and pollen fertility. In *cals5* mutants, the exine wall organization is also disrupted with malformed baculae and tectum as well as randomly deposited globular tryphine, showing that the callose wall is required for exine sculpting (Dong et al., 2005). Similarly, a knockdown or a knockout of rice Glucan Synthase-Like 5 (GSL5) reduces callose production during pollen development, compromising exine deposition and pollen fertility (Shi et al., 2015b). The timely degradation of callose is also required for pollen fertility. Rice plants silenced for the β-1,3-glucanase gene *Osg1* have delayed release of the microspores from the tetrad, leading to their degeneration and male sterility (Wan et al., 2011).

Several mutants also suggest an important role for pectin throughout pollen development (Suárez-Cervera et al., 2002; Phan et al., 2011; Chebli et al., 2012; Jiang et al., 2013; Cankar et al., 2014). The major form of pectin, homogalacturonan (HG, β-1, 4-galacturonic acid), is initially synthesized in the Golgi in a methyl-esterified form (meHG). After secretion, the methyl groups may be enzymatically removed by pectin methyl-esterases (PMEs) to form de-methyl-esterified HG (dmeHG), exposing negative charges, which can form Ca^2+^ salt bridges between neighboring polymers, rigidifying the wall (Bosch and Hepler, 2005). De-methyl-esterification can also promote pectin degradation by polygalacturonases (PGs) (Verlag et al., 2003). The separation of the microspores at the tetrad stage requires pectin remodeling as evidenced by the Arabidopsis *quartet* mutants (*qrt1, qrt2*, and *qrt3*) in which the pollen completes development as a tetrad (Preuss et al., 1993; Rhee et al., 2003). *QRT1* encodes a PME and *QRT2* and *QRT3* encode PGs (Aouali et al., 2001; Rhee et al., 2003; Francis et al., 2006; Ogawa et al., 2009). Pectin remodeling is also important for the formation of the intine as demonstrated by Arabidopsis *pme48* mutants in which the intine accumulates high levels of meHG, resulting in defective pollen imbibition and germination defects (Leroux et al., 2015). In Chinese cabbage, co-inhibition of two closely related PGs, Brassica campestris Male Fertility 26a (BcMF26a) and BcMF26b, has defective pollen intine and severely inhibited male fertility (Lyu et al., 2015).

While broad landmarks of anther and pollen development are well-known and generally conserved, the molecular events of PG wall formation have received much more limited attention, particularly with respect to the development of the intine (Gómez et al., 2015; Ma et al., 2021). Here, we focus on elucidating this process during tomato pollen development. Tomato is an important agricultural crop, which requires successful pollination for a fruit set (Picken, 1984). It is also highly sensitive to environmental disruptions of fertility, particularly high-temperature stress (Pressman et al., 2002; Giorno et al., 2010, 2013; Müller et al., 2016). Therefore, understanding the development of pollen is an important preliminary step in safeguarding production against environmental insults. Tomato also serves as a model system for the large Solanaceae family, which includes many other agriculturally important plants. Given the importance of the pollen grain wall to pollen fertility, we have established a timeline of polymer deposition and remodeling during tomato pollen grain wall formation.

## Materials and Methods

### Plant Material and Growth Conditions

*Solanum lycopersicum* cv. Micro-Tom (Carvalho et al., 2011) was grown under 16-h light:8-h-dark cycles in temperature-controlled growth chambers maintained at 23°C. Buds were staged by measuring the length from the bud tip to the pedicle with a vernier caliper. For scanning electron microscopy (SEM) of pollinated pistils, anthers were removed from 6-mm flower buds, and pistils were covered with open 0.2-ml PCR tubes and allowed to mature for 48 h. Pollen was collected by vortexing freshly collected mature anthers in 1.5-ml epitubes and applied to pistils with a paint brush. Pollinated plants were returned to the growth chamber, watered, and covered with a clear plastic dome to increase humidity. Five hours after pollination, pistils were fixed and treated for SEM as described below.

### Scanning Electron Microscopy

For scanning electron microscopy (SEM), manually pollinated pistils were collected and submerged in fresh fixative (acetic acid/ethanol, 1:3), and fixed under a vacuum for 2 h. Pistils were then transferred to fresh 100% ethanol and kept at 4°C overnight in a tightly closed glass vial. The tissue was then dehydrated, using chemical drying with graded hexamethyldisilazane (HDMS) (Sigma-Aldrich catalog number 440191) series (Lee and Chow, 2012). Ethanol was slowly replaced with HDMS for 20 min each in ethanol: HDMS series of 1:0, 3:1, 3:2. 1:1, and 1:3, and three times 100% HDMS. Afterwards, tissue was left overnight in the HDMS in an open tube under the fume hood. In the morning, the HDMS evaporated, and the tissue was dry and ready for gold coating. Tissue was attached to SEM stubs with double-sided tape, and sputter coated with gold particles in a vacuum at 200-m Amps for 120 s (~15–20 nm of gold), using a Denton Desk II sputter coater. For imaging of pollen grains, dry mature pollen grains were collected in epitubes from healthy, mature dehiscent flowers. Pollen was sprinkled on adhesive SEM stubs with paint brush bristles and sputter coated with gold particles in a vacuum for 90 s. Gold-coated pollen and pistils were imaged, using the EMAL JEOL JSM-7800FLV field-emission scanning electron microscope. For pore diameter measurements, three random pictures, each of four individual pollen grain, were taken, and diameters of all the pores in each micrograph were measured. The shown values are average across all measurements.

### Transmission Electron Microscopy

Pollen grains were suspended in ice-cold fixative, containing 2% formaldehyde, 2.5% glutaraldehyde, .025-M PIPES buffer pH 7.2, and 0.001% Tween 20 *via* vortexing. After 2-h incubation under a vacuum, the solution was replaced with fresh fixative, and vials were sealed and kept at 4°C overnight. Pollen was then pelleted by centrifugation at room temperature and washed with 0.025-M phosphate buffer pH 7 for 10 min, followed by a PIPES buffer pH 7.2 wash for 20 min. Pollen was then post fixed in 1% osmium tetroxide (Sigma-Aldrich catalog number 75633) in phosphate buffer pH 7 for 1 h. This was followed by another 10-min phosphate buffer wash, followed by 20-min PIPES buffer wash. Pollen was then dehydrated for 20 min each in an ethanol series (25, 35, 50, 70, 80, 90, and three times 100%). Ethanol was then replaced slowly with an LR white plastic polymer (Agar Scientific catalog number AGR 1281) by an LR white: ethanol series of 1:3, 2:3, 1:1, 3:2, 3:1,1:0, 1:0, and 1:0, with each step of the series having a 4°C incubation of 24 h. At this stage, pollen was put in a gelatin capsule with fresh LR white and polymerized at 58°C for 24 h. Solid polymer blocks were sectioned into 80-nm ultrathin sections and stained with 7% uranyl acetate and lead citrate. Sections were imaged with the JOEL JEM-1400 plus electron microscope with an XR401 SCMOS camera. For cell wall measurements, three pollen grains were measured at 10 random positions per grain, excluding the apertures; the average across all measurements was taken.

### Paraffin Embedding, Sectioning, and Toluidine Blue Staining

To identify appropriate bud length for each pollen development stage, flower buds of 2, 4, 5, 6, 8-mm, and fully mature dehiscent flowers were fixed by submerging in ice cold fixative (4% PFA and 0.3% Tween20 in PBS) and vacuum infiltrated for 20 min. Fresh fixative and a vacuum were applied until the tissue sunk to the bottom. The tissue was then dehydrated in a PBS-ethanol series of 10, 30, 50, 70, 85, and 100% ethanol for 1 h each at 4°C. The tissue was then infiltrated with histoclear (Avantor, Electron Microscopy sciences, catalog number 101412-878) in ethanol: histoclear series (3:1, 1:1, 0:1, 0:1, and 0:1) at room temperature. Histoclear was then slowly replaced with molten paraffin at 60°C by replacing the histoclear with a histoclear: paraffin series (3:1, 1:1, 1:3, 0:1, 0:1, and 0:1) for 24 h each at 60°C. Final paraffin-embedded tissue was sectioned with a microtome to 8-μm thick sections and placed on polylysine-coated slides. Sections were dewaxed by submerging in histoclear and dehydrated by dipping in an ethanol series (10, 30, 50, 70, 85, and 100%) ethanol in PBS. Dewaxed and dehydrated sections were stained with 0.1% toluidine blue (Cankar et al., 2014) by applying the stain and heating the slide to 60°C for 30 s. After stain application, slides were washed with distilled water and permanently mounted by applying Permount (Fischer Scientific catalog number SP15-500) and cover slip.

### LR White Embedding, Semi-thin Sectioning, and Immunostaining

Immunohistochemistry analysis was done as previously described (Luis da Costa et al., 2017). Briefly, flower buds of the appropriate length were removed from the plant, and, for buds bigger than 2 mm, the anthers were dissected out. The tissue was fixed and LR white embedded as described for TEM above, without the osmium tetroxide post-fixation step. Hardened samples were sectioned with glass knife ultramicrotome in 200-nm semi-thin sections. Sections were observed and imaged with light microscopy by staining with 0.1% toluidine blue in the water. For calcofluor white staining, one drop of calcofluor white solution (containing Calcofluor White 1 g/l, Evans blue 0.5 g/l; Sigma-Aldrich, 18909) was added to the well of the slide and imaged after applying the coverslip. For aniline blue staining of callose, one drop of aniline blue fluorochrome (0.1-mg/ml in distilled water; Biosupplies, 18,909) was applied to the well of the slide and imaged after applying the coverslip. For immunostaining, sections were placed in welled 0.001% poly-L-Lysine-coated slides. After drying at 50°C overnight, sections were incubated in blocking solution (5% non-fat dried milk in 1x PBS) for 10 min in a humid chamber. Then the sections were washed for 10 min with PBS. Sections were incubated at room temperature for 2 h, followed by 4°C incubation overnight in primary antibody solution (Rat monoclonal LM19 or LM20; a 1:5 antibody in blocking solution; Verhertbruggen et al., 2009). Sections were then washed two times with PBS for 10 min and then incubated with secondary antibody solution (Anti-Rat conjugated with FITC, Sigma-Aldrich, F6258; 1:100 in blocking solution) for 3–4 h at room temperature in the dark. Sections were then washed two times with PBS for 10 min each. Sections were imaged after adding one drop of calcofluor white and applying a drop of Vectashield® antifade (Vector laboratories catalog number H-1000-10) and applying the coverslip. Sections were imaged, using the Leica SP5 laser scanning confocal microscope.

## Results

### The Structure of Mature Tomato Pollen

**Figure 1:**
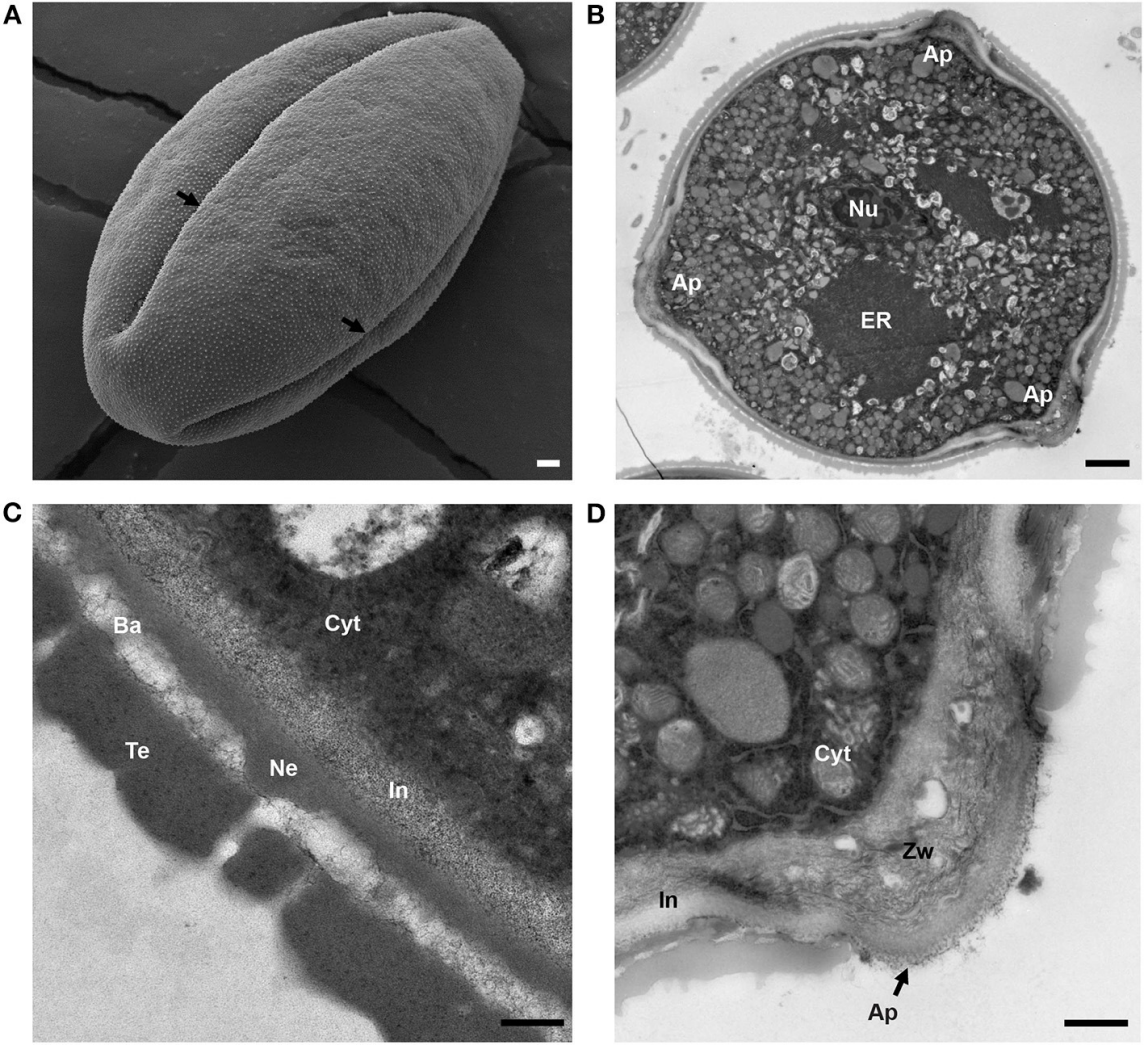
Structure of mature tomato pollen grain. **(A)** Scanning electron micrograph of a mature, released tomato pollen grain. Arrows mark the apertures (Ap). **(B)** Transmission electron micrograph of a mature pollen grain cross section. Nu, nucleus; ER, endoplasmic reticulum; Ap, aperture. **(C)** TEM of mature pollen, showing the layers of the pollen wall. Cyt, cytoplasm; In, intine; Ne, nexine; Ba, bacula; Te, tectum. **(D)** TEM close-up of pollen aperture. Arrow marks the aperture. Cyt, cytoplasm; In, intine; Zw, zwischenkorper. Scale bars 1 μm in **(A)**, 2 μm in **(B)**, 200 nm in **(C)**, and 600 nm in **(D)**.

To observe the general shape and structure of the tomato pollen, we imaged mature-released pollen grains, using scanning electron microscopy (SEM). SEM micrographs showed that the tomato pollen exhibits features of a typical dicot pollen grain, with a prolate spheroid shape, containing three radially equidistant elongated apertures (Figure 1A). The outer surface of the pollen was textured with spines of exine interspersed with small pores with an average diameter of 38 ± 9 nm (Supplementary Figures 1B–D).

To observe the internal morphology of the pollen grain, we used transmission electron microscopy (TEM) to image transverse sections of the mature pollen grain (Figure 1B). Inside, the pollen was populated with an extensive ER network, vegetative and generative nuclei, numerous mitochondria, vacuoles as well as starch granules. In some sections, Golgi stacks were also visible. Upon close-up examination of the pollen wall structure (Figure 1C), intine was apparent as the inner, lighter, less electron dense layer around the pollen compared with the outer exine, which stained much darker. The layered organization of the exine was also apparent with the nexine layer directly adjacent to the intine, followed by the columnal baculae and, finally, the outermost tectum layer (Figure 1C). We measured the thickness of the overall pollen wall (658.54 ± 58 nm) and the cell wall layers (intine 151 ± 28 nm, sexine 398 ± 38 nm, nexine 112 ± 30 nm). The intine wall was continuous around the perimeter of the pollen grain, but exine was absent at the apertures. At the apertures, while the exine was absent, the inner layer of intine formed a curved outward projection at the aperture site, called the “Zwischenkörper.” The intine at this site was thickened and with a layered and spongy appearance (Figure 1D).

### The Pollen Development Stage Correlates With Flower Bud Length

**Figure 2:**
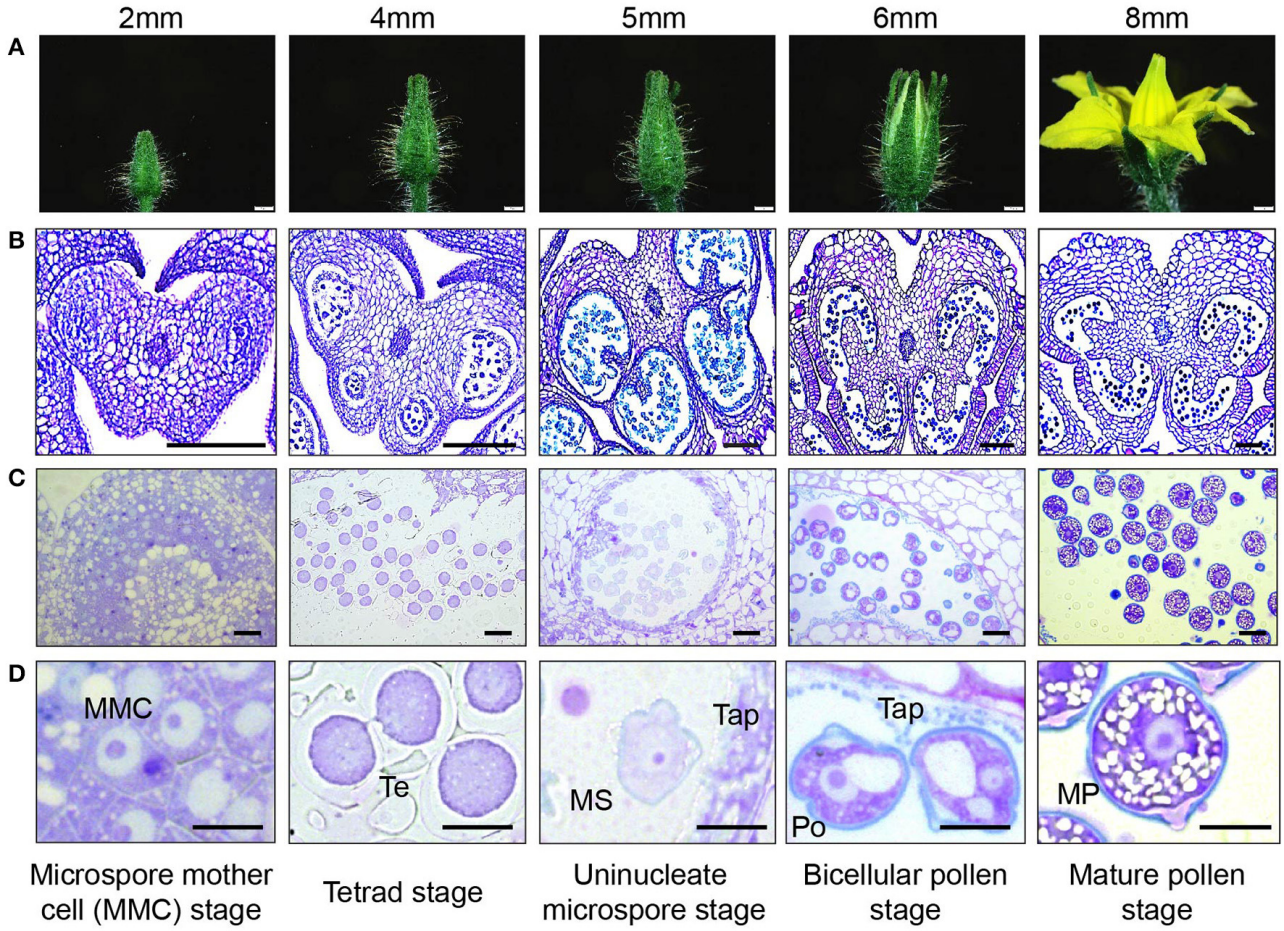
Tomato pollen development staging. **(A)** photographic series of floral development in tomato, with bud length as a marker for each stage in pollen development inside the anther. Bud lengths, 2 mm (microspore mother cell stage), 4 mm (tetrad stage), 5 mm (uninucleate microspore stage), 6 mm (bicellular pollen stage), and 8 mm (mature pollen stage). **(B)** Micrographs of paraffin-embedded 8-μm-thick anther cross sections, stained with toluidine blue. **(C)** Micrographs of LR White embedded 500-nm-thick anther cross sections, also stained with toluidine blue. **(D)** Developing pollen grain at higher magnification, close-up of individual cells. MMC, microspore mother cell; Te, tetrad; MS, microspore; Tap, tapetum; Po, pollen; MP, mature pollen. Scale bars 1 mm in **(A)**, 100 μm in **(B)**, 20 μm in **(C)**, and 10 μm in **(D)**.

To establish a reproducible staging method for pollen development, we compared the morphology of anthers and developing pollen in toluidine blue-stained sections of floral buds at various bud sizes. We found that flower bud length correlated well with developmental stages of the pollen grain and could be effectively used to non-destructively estimate the developmental stage of the pollen (Figure 2A).

In the anthers of 2-mm-long buds, the microspore mother cells (MMCs) were visible with enlarged nuclei (Figures 2B–D). Cells in the center of the anther were continuous at this stage without locular pockets (Figure 2B, Supplementary Figure 2A). At the 4-mm bud stage, meiosis was complete, and the meiotic products were still joined in a tetrad. The tetrad cells had distinct centrally located nuclei, with no obvious vacuoles or visible nucleolus. The thick pollen wall did not stain with toluidine blue at this stage, but its outer edge was visible in sections (Figures 2B–D). In the anther, there was a distinct tapetal cell layer surrounding four locular pockets, larger and smaller pockets in each anther lobe (Supplementary Figure 2B).

Pollen in the 5-mm flower buds was at the early microspore stage. The microspores had an ameboid shape, and their nuclei had a distinct outline and visible nucleolus (Figures 2B–D). In the anthers, the locules on each side of the anther considerably enlarged but were still separated by interlocular septa and were still lined with a distinct visible tapetum cell layer (Supplementary Figure 2C). By this stage, the non-staining tetrad wall had disappeared. In the 6-mm flower bud, the first pollen mitosis occurred, producing bicellular pollen with a more rounded shape and a large central vacuole. The anthers were yellow, and the two locule pockets on each side had joined to make one continuous locule cavity after the deterioration of interlocular septa (Supplementary Figure 2D). The tapetum had also broken down, with the leftover vestiges visible in the locular cavity and around the pollen.

Pollen in the 8-mm flower was fully mature, with a well-rounded shape and one or two nuclei visible in sections. In tomato, the second pollen mitosis, producing the two sperm nuclei, occurs during pollen tube elongation (Brukhin et al., 2003). The pollen at this stage also had a distinct, two layered wall differentially stained with toluidine blue. The purple inner layer was especially prominent at the aperture sites. The pollen also contained abundant, unstained starch granules (Figures 2B–D). The tapetum fully disappeared. The flower was open, and the anther was close to dehiscence upon breakage of the weak anther stomium (Figure 2A).

### Exine Deposition Occurs Post Tetrad

**Figure 3:**
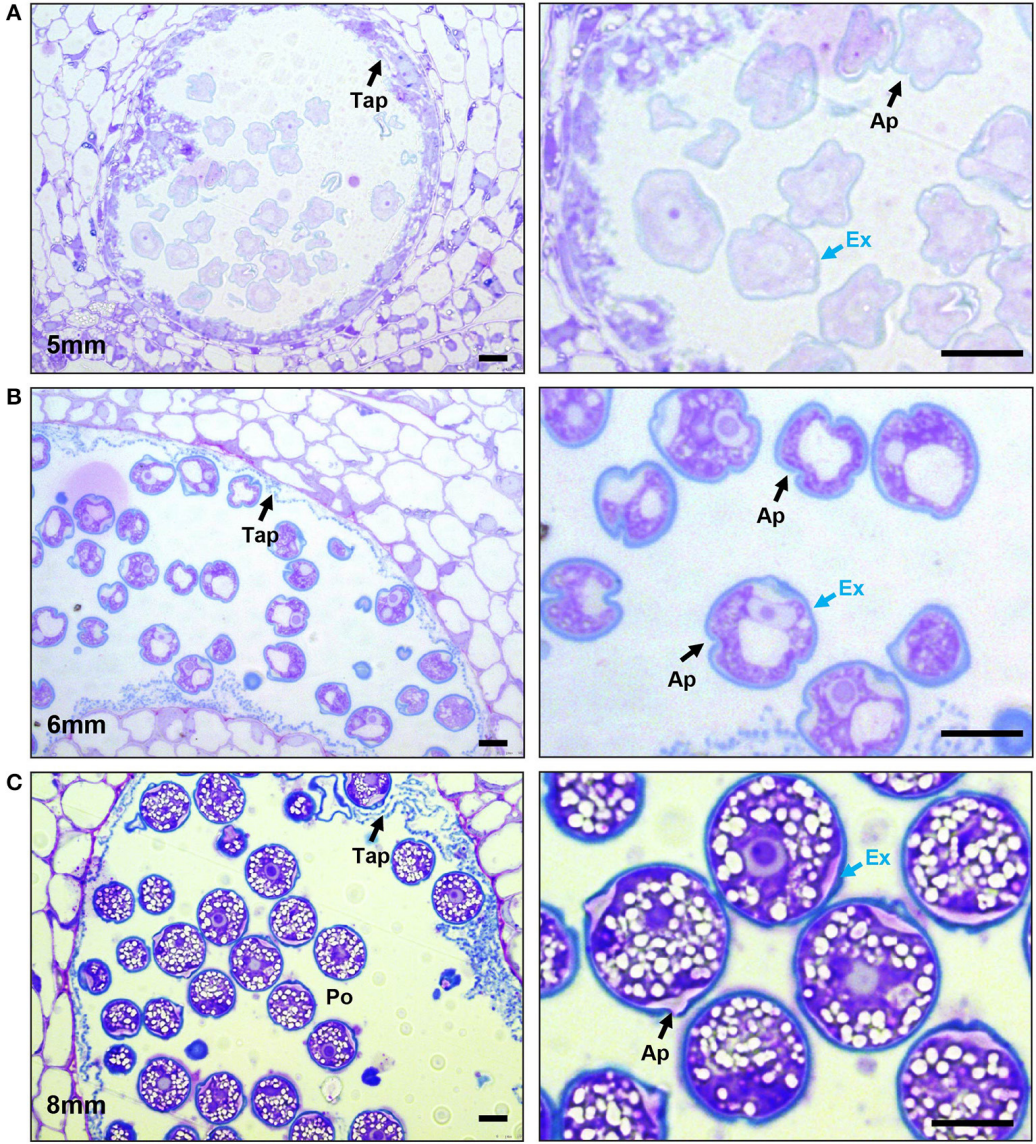
Progression of exine deposition. Micrographs of LR White embedded 500-nm thick anther cross sections, stained with toluidine blue. **(A)** The uninucleate microspore stage at 5-mm bud length. Cross section of anther locule (Left), The arrow marks the tapetum. Close-up of microspores (right), black arrow marks aperture; the blue arrow marks the exine. **(B)** The bicellular pollen stage at 6-mm bud length. Cross section of anther locule (left); the arrow marks the tapetum. Close-up of microspores (right), black arrow marks aperture; the blue arrow marks the exine. **(C)** The mature pollen stage at 8-mm bud length. Cross section of anther locule (left); the arrow marks the tapetum. Close-up of microspores (right); the black arrow marks aperture; the blue arrow marks the exine. Ex, exine; Tap, tapetum; Po, pollen; Ap, aperture. All scale bars are 10 μm.

Sporopollenin is a distinct biological polymer, composed of fatty acid derivatives and phenolic compounds (Piffanelli et al., 1998; Blackmore et al., 2007). It is only found in the exine layer of the pollen grains and plant spores and is one of the most resilient biological molecules. Toluidine blue (TD) is a basic thiazine metachromatic dye and differentially stains acidic residues with high affinity (Sridharan and Shankar, 2012). Since sporopollenin is acidic, it stains differently with toluidine blue stain (bluer as compared with intine, which stains purple) (Dobritsa et al., 2011). We used toluidine blue-stained sections to observe exine deposition in developing pollen. After no cell wall staining with TD in the tetrad stage (4-mm buds), a thin layer of exine was visible in the early microspores (a 5-mm bud) (Figure 3A). This foot layer had visible breaks at the slightly invaginated future aperture sites (Figure 3A, right). In the bicellular pollen stage (a 6-mm bud), the exine was much thicker, indicated by a thicker, bluer pollen outline, and the aperture sites were visible as indentations in the exine (Figure 3B). By this stage, tapetum had degenerated, leaving behind pollenkitt and pollen coat particles. In the 8-mm buds, the mature pollen had a solid blue outline, showing a fully developed exine wall, as well as a differentially stained intine also clearly visible (Figure 3C).

### Cellulose and Callose Dynamics

While certain cell wall changes were apparent in sections stained with toluidine blue, the status of other cell wall polymers required further investigation. Therefore, to follow pollen wall development, we stained anther sections of the defined developmental stages with various carbohydrate-binding dyes and cell wall antibodies. The fluorescent dye Calcofluor white is a fluorochrome with a high affinity for cellulose (β-1,4-glucan), and chitin and is regularly used to observe pollen intine, although it also possesses some affinity for callose (Fang et al., 2008; Herburger and Holzinger, 2016; Li et al., 2017; Renzaglia et al., 2020; Takebe et al., 2020). Aniline blue fluorochrome (ABF) is a callose-specific dye often used to stain pollen tube cell walls (Fang et al., 2008; Herburger and Holzinger, 2016; Renzaglia et al., 2020).

**Figure 4:**
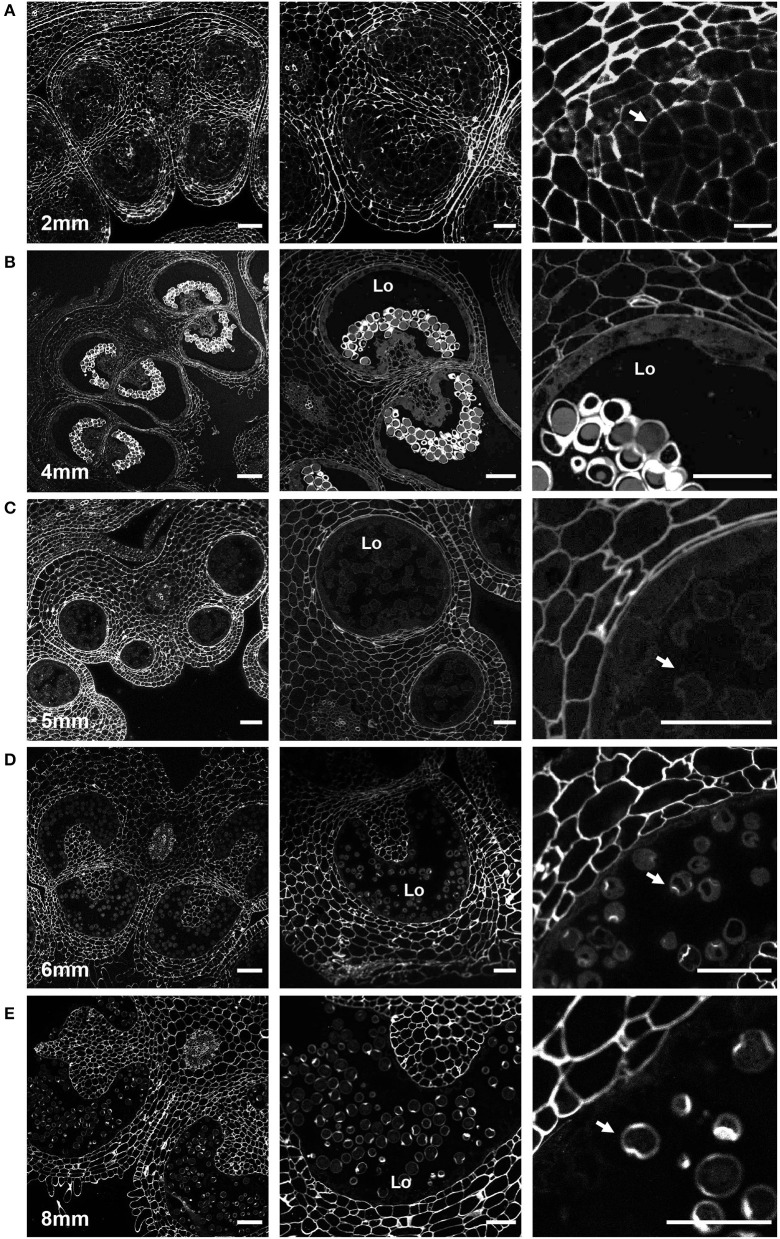
Calcofluor white staining of pollen development. Fluorescent micrographs of anther cross sections (500 nm) in LR white, stained with calcofluor white. **(A)** The microspore mother cell stage in the 2-mm bud anther. **(B)** The tetrad stage in the 4-mm bud anther. **(C)** The uninucleate microspore stage in the 5-mm bud anther. The arrow marks the microspore wall. **(D)** Bicellular pollen in 6-mm bud anther. The arrow marks the pollen wall. **(E)** Mature pollen in 8-mm bud anther. The arrow marks pollen wall. Te, tetrad. Scale bars. **(A)** 50 μm (left), 25 μm (center and right). **(B)** 75 μm (left), 50 μm (center and right). **(C)** 50 μm (left), 25 μm (center and right). **(D)** 75 μm (left), 50 μm (center and right). **(E)** 75 μm (left), 50 μm (center and right).

**Figure 5:**
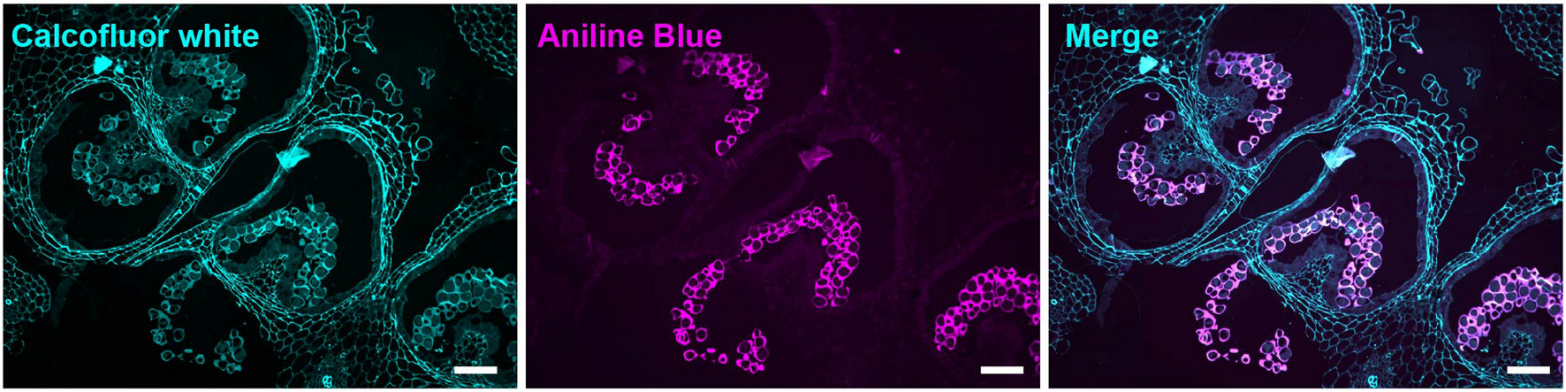
Aniline blue staining of the tetrad callose wall. Fluorescent micrographs of anther cross sections (500 nm) in LR white, of the 4-mm bud anther, stained with calcofluor white and aniline blue fluorochrome. Calcofluor white staining (cyan) (left). Aniline blue staining (magenta) (center). Merged staining (right). Scale bars 50 μm.

In 2-mm buds, calcofluor white weakly stained the MMC cell walls but strongly stained the cell walls of the surrounding anther tissue, suggesting reduced cellulose content in the MMC walls (Figure 4A). In 4-mm buds, the tetrad-stage pollen wall stained strongly with both calcofluor white (Figure 4B) and ABF (Figure 5). ABF did not stain the pollen or the surrounding anther tissue during any other stage of development (not shown). After dissolution of the transient callose wall, in 5-mm buds, no calcofluor white signal was observed around the uninucleate microspore (Figure 4C), although light microscopy at this stage showed there is a thin wall around the pollen (Figure 2D). In the 6-mm bud, the bicellular pollen had a thin rim of calcofluor white signal but a robust signal at the aperture sites, consistent with the development of the thick intine of the Zwischenkörper (Figure 4D). In the mature pollen in the 8-mm flower bud, the pollen had a continuous calcofluor white-positive layer around the perimeter of the pollen, with particularly prominent signal at the apertures (Figure 4E).

### Pectin Dynamics

To examine the pattern of meHG and dmeHG during pollen development, we immuno-stained anther sections with rat monoclonal antibodies LM20 and LM19 (Verhertbruggen et al., 2009). LM19 preferentially binds HG with a lower degree of esterification (i.e., deHG), while LM20 shows a complementary affinity, binding to more heavily methylated HG, although a degree of overlap does exist in their binding abilities (Christiaens et al., 2011). As a preliminary to the use of these antibodies, we first demonstrated that the fluorophore-conjugated secondary antibody did not bind to our anther sections (Supplementary Figure 3).

**Figure 6:**
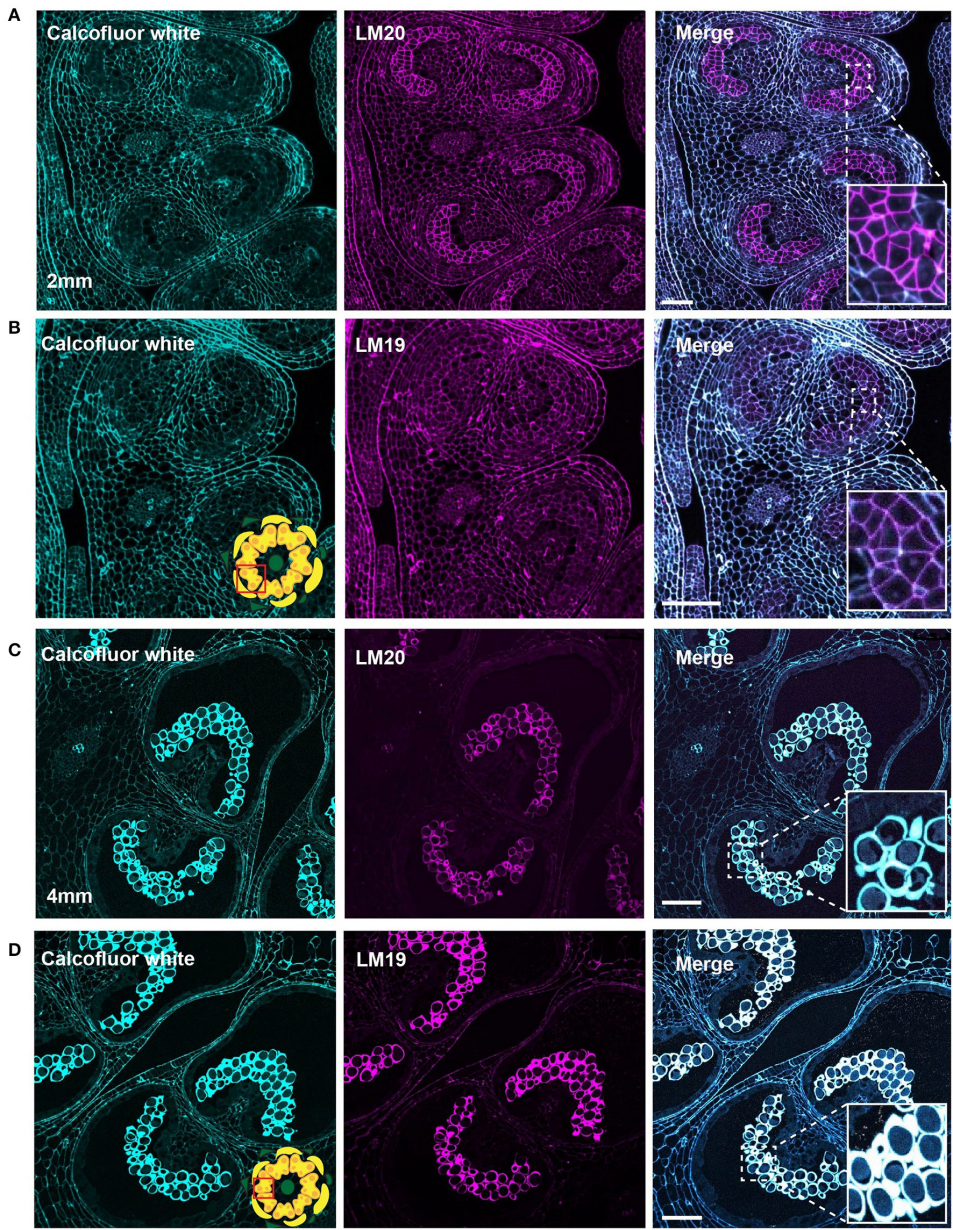
LM20 and LM19 immunostaining of the 2-mm and 4-mm bud anthers. Fluorescent micrographs of anther cross sections (500 nm) in LR white, of the 2-mm and 4-mm bud anther, stained with Calcofluor white and FITC conjugated anti-rat secondary for LM20 or LM19. Calcofluor white staining (Cyan) (left). Aniline blue staining (magenta) (center). Merged staining (right). **(A)** LM20 staining of the 2-mm anther. The right panel inset, showing zoomed-in view of the MMCs wall with the merged staining. **(B)** LM19 staining of the 2-mm anther. The right panel inset, showing zoomed-in view of the MMCs with the merged staining. **(C)** LM20 staining of the 4-mm anther. The right panel inset, showing zoomed-in view of the tetrad cells with the merged staining. **(D)** LM19 staining of the 4-mm anther. The right panel inset, showing zoomed-in view of the tetrad cells with the merged staining. Scale bars 50 μm.

In the 2-mm bud, while the MMCs had a weak calcofluor signal, indicating low levels of cellulose, the LM20 signal was robust and generally stronger than that of the surrounding anther tissue (Figure 6A). Close-up observation of the MMC walls showed boundaries of each cell thickly marked with LM20. To observe the pattern of dmeHG at this stage, we also stained with LM19. While the boundaries of MMCs did stain with LM19, the signal was considerably less robust than that of LM20, and, generally, the MMC signal was consistent with the signal observed for the cell wall of the surrounding anther cells (Figure 6B).

**Figure 7:**
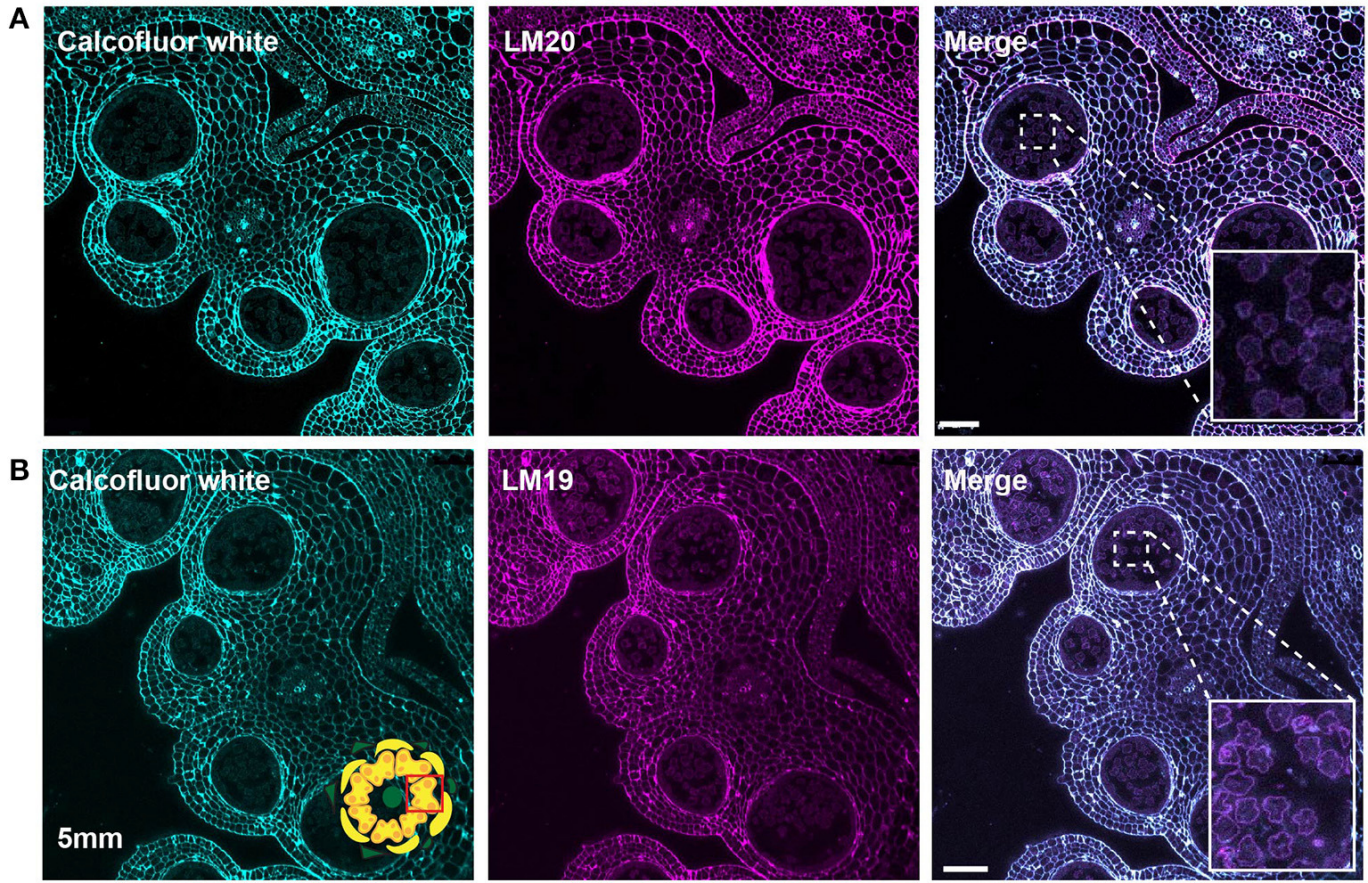
LM20 and LM19 immunostaining of the 5-mm bud anthers. Fluorescent micrographs of anther cross sections (500 nm) in LR white, of the 5-mm bud anther, stained with Calcofluor white and FITC conjugated secondary for LM20 or LM19. Calcofluor white staining (Cyan) (left). Aniline blue staining (magenta) (center). Merged staining (right). **(A)** LM20 staining of the 5-mm anther. The right panel inset, showing zoomed-in view of the microspore wall with the merged staining. **(B)** LM19 staining of the 5-mm anther. The right panel inset, showing zoomed-in view of the microspore with the merged staining. Scale bars 50 μm.

At the tetrad stage in the 4-mm bud, the callose wall also stained positive for the LM20 and LM19 epitopes (Figures 6C,D). At this stage, the LM19 signal was more robust, and the tetrad walls were strongly stained relative to other anther cells (Figure 6D). Therefore, the pollen wall at the tetrad stage contained abundant meHG and dmeHG in addition to callose (Figure 5). However, at the early microspore stage in 5-mm buds, pectin distribution was largely altered. meHG, as detected by LM20 binding, was considerably reduced with only a faint signal detectable around the microspores, although the surrounding anther tissue stained robustly (Figure 7A). The LM19 signal at this stage was similarly reduced compared with the tetrad stage, but a clear outline of the microspore was visible and was more robust than the corresponding LM20 signal (Figure 7B). Therefore, the loss of the callose wall coincided with a major loss of pectin during the transition between the tetrad and microspore stages.

**Figure 8:**
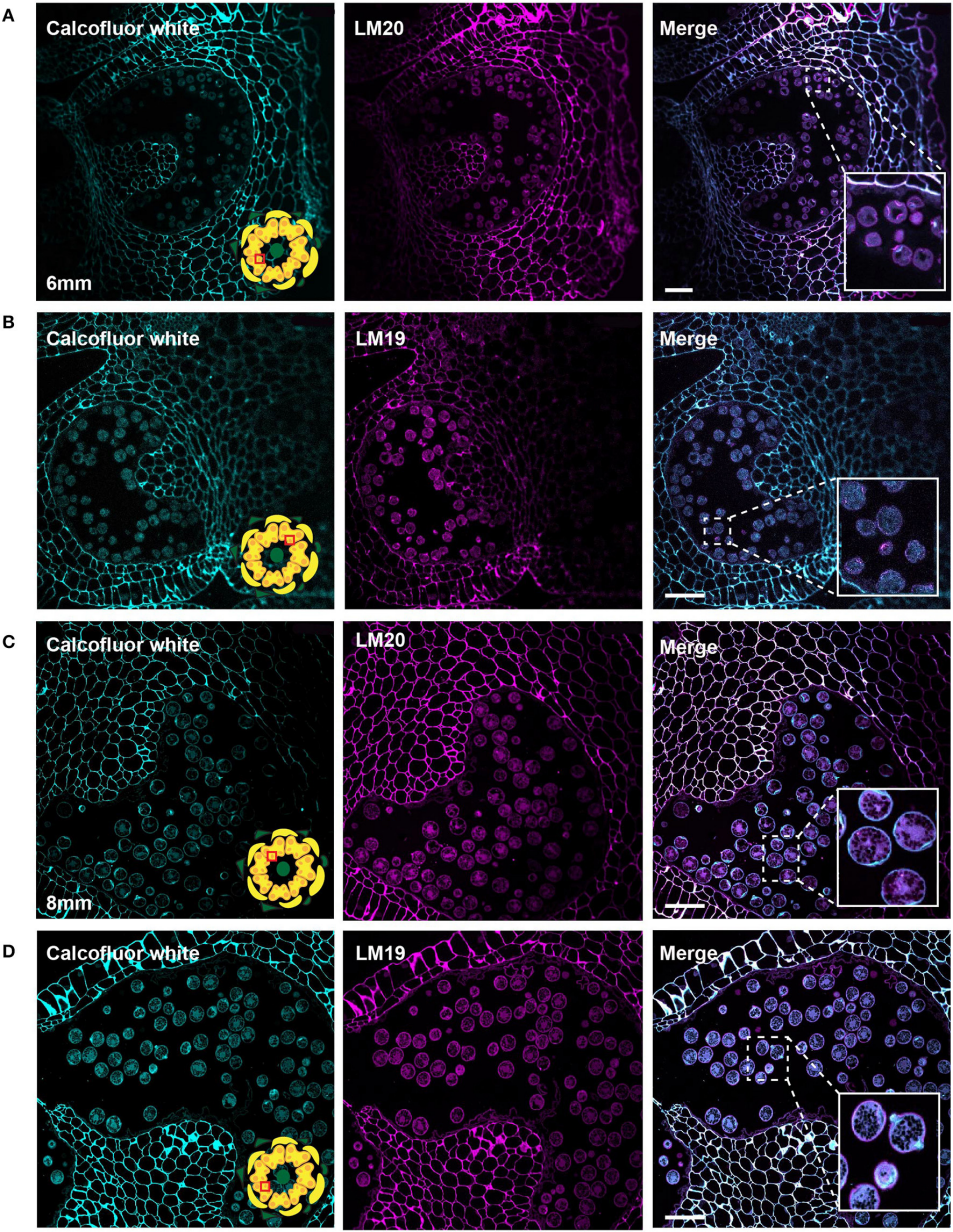
LM20 and LM19 immunostaining of the 6-mm and 8-mm bud anthers. Fluorescent micrographs of anther cross sections (500 nm) in LR white, of the 6-mm and 8-mm bud anther, stained with Calcofluor white and FITC conjugated anti-rat secondary for LM20 or LM19. Calcofluor white staining (cyan) (left). Aniline blue staining (magenta) (center). Merged staining (right). **(A)** LM20 staining of the 6-mm anther.The right panel inset, showing zoomed-in view of the pollen wall with the merged staining. **(B)** LM19 staining of the 6-mm anther. The right panel inset, showing zoomed-in view of the pollen with the merged staining. **(C)** LM20 staining of the 8-mm anther. The right panel inset, showing zoomed-in view of the pollen wall with the merged staining. **(D)** LM19 staining of the 8-mm anther. The right panel inset, showing zoomed-in view of the pollen wall with the merged staining. Scale bars 50 μm.

In the bicellular stage, pollen in the 6-mm bud, we observed a diffuse LM20 signal throughout the pollen. In addition, we also found a robust signal at the inner surface of the aperture sites (Figure 8A). The LM19 signal was similarly diffuse in the pollen grain but was not enriched at the aperture sites at this stage (Figure 8B). In the mature pollen, in the 8-mm buds, there was a distinct boundary around the pollen with LM20 signal (Figure 8C) and a similar, though the more robust boundary of the LM19 signal (Figure 8D). The mature apertures sites were robustly stained with calcofluor white at this stage but were generally deficient in the LM19 and LM20 signals relative to the non-aperture regions of the pollen wall.

## Discussion

**Figure 9:**
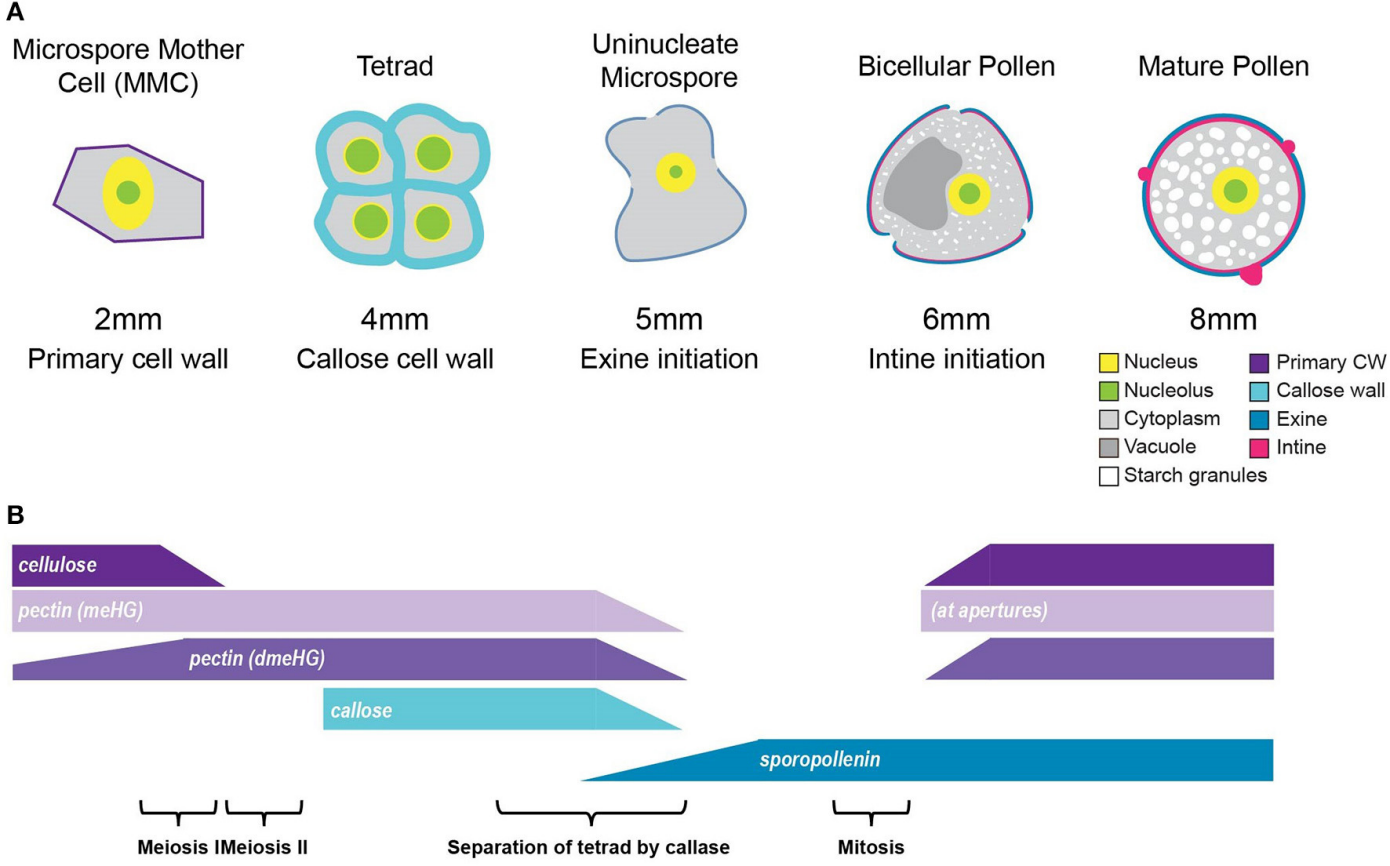
Summary of tomato pollen wall development process. **(A)** A graphic summary of the major hallmarks of the tomato pollen development timeline. The pollen development stage is given above, and the bud length corresponding to each stage is given below, along with the major cell wall identities. **(B)** Cell wall polymer changes during development. The cellulose of the primary cell wall of the MMC breaks down at the 2-mm bud stage. Following meiosis, the tetrad cells are surrounded by a thick callose and pectin wall, which is broken down to separate the tetrad cells into free uninucleate microspores. Exine deposition by the tapetal cells begins at the 5-mm bud stage and continues through pollen maturation. The deposition of the cellulose and pectin, which make up the intine, initiates at the 6-mm bud stage and also continues through maturation. meHG deposition at the bicellular pollen stage is initially limited to the aperture regions, later spreading to the whole intine.

The transition from the general cell wall of a non-specialized anther cell to the structurally distinct wall of the mature pollen grain requires considerable remodeling. Here, we have combined imaging techniques to observe the structure of the tomato pollen wall and to establish the sequence of cell wall polymer remodeling events taking place during pollen development, beginning with the MMCs and continuing to pollen maturation (Figure 9). In the premeiotic anthers of 2-mm buds, we observed significant differences in cell wall composition between the MMCs and the surrounding anther cells. Weak calcofluor white staining (Figure 4A) and the robust LM20 signal (Figure 6A) in the cell walls of the MMCs are consistent with a reduction in cellulose and higher levels of meHG in these cells compared with other anther cells. These observations support the interpretation of previous tomato anther TEM data, which described an approximately 10-fold expansion of the width of the middle lamella between premeiotic MMC and those in early prophase and the loss of microfibrils adjacent to the plasma membrane by late prophase (Polowick and Sawhney, 1992). Reduced cellulose, combined with increased meHG, would be consistent with a more extensible cell wall, possibly facilitating the expansion and remodeling required for further pollen development. Therefore, even in this early stage of pollen development, the cell wall composition of the pollen lineage cells was already distinct.

At the tetrad stage in 4-mm buds, a thick callose wall surrounding the postmeiotic tetrads was apparent in ABF-stained sections (Figure 5). Unlike cellulose, which is a common plant cell wall component, callose occurs transiently during cell division and in some specialized cell types or under specific conditions (Chasan, 1992; Mollet et al., 2013; Renzaglia et al., 2020). The tetrad stage was the only stage at which we observed callose, and it was limited to the tetrad wall. While the ABF signal, indicating the presence of callose was clear, the observation of calcofluor white binding at this stage (Figure 4B) must be interpreted with caution as it may indicate the presence of cellulose or might result from non-specific binding to callose. In terms of pectin content, we observed both LM20 and LM19 signals, indicating that the tetrad wall also contained HG of low and high degrees of methylesterification (Figure 6B). Furthermore, enhancement of the LM19 signal in the tetrad wall suggests that dmeHG might serve a significant functional role here, possibly providing stiffness to the callose wall. Lack of aniline blue staining in the 5-mm bud anther demonstrated that the callose wall is temporary and after meiosis degrades to separate the tetrads into individual microspores (Polowick and Sawhney, 1993a; Owen and Makaroff, 1995; Paxson-Sowders et al., 1997; Kravchik et al., 2019; Zhang et al., 2020). At this stage, most of the LM19 and LM20 signals were also lost (Figure 7). These observations are also consistent with the effect of the Arabidopsis *qrt* mutants and their disruption of pectin remodeling enzymes, blocking tetrad separation (Preuss et al., 1993; Aouali et al., 2001; Rhee et al., 2003; Francis et al., 2006; Ogawa et al., 2009). The calcofluor white signal was also weak in the early microspore stage (Figure 4C), indicating little cellulose. We also detected a thin layer of exine at this stage, which may further support the wall structure (Figure 3). The primexine has an important function during this stage; unfortunately, the composition of the primexine is poorly understood, making it difficult to study (Li et al., 2017; Wang and Dobritsa, 2018). Other possible microspore cell wall components include cell wall-associated proteins, for example, the extensins or arabinogalactan glycoproteins (AGPs). The extensins are moderately glycosylated, highly repetitive proteins, which can be covalently cross-linked in the wall by secreted peroxidases, possibly forming a scaffold for pectin assembly (Schnabelrauch et al., 1996; Cannon et al., 2008; Petersen et al., 2021). In Arabidopsis *peroxidase9* (*prx9*) and *prx40* double mutants, microspores and tapetal cells have a swollen phenotype, suggesting compromised cell wall integrity due to a reduction in extensin cross-linking during pollen development (Jacobowitz et al., 2019). Evidence for possible AGP involvement at this stage of pollen development comes from *UNEQUAL PATTERN OF EXINE1/KAONASHI4* (*UPEX1/KNS4*), a β-(1,3)-galactosyltransferase responsible for the synthesis of the AG glycans found on AGPs and rhamnogalacturonan I, a pectic polysaccharide. *upex1/kns4* mutants have lower levels, and an altered pattern of AGPs at the microspore stage and disrupted primexine formation and exine patterning (Dobritsa et al., 2011; Li et al., 2017; Suzuki et al., 2017).

The aperture sites began to take on a distinct cell wall identity by the bicellular stage (6-mm buds) when the cytoplasmic face of the future aperture sites accumulates cellulose and meHG as indicated by the LM20 signal (Figure 8A). The accumulation of LM20 at this location suggests delivery of newly synthesized meHG through targeted exocytosis to these sites. By the mature stage, the apertures had a complex organization with a calcofluor-positive inner core and a ring rich in LM19 and LM20 epitopes visible in some pollen sections (Figures 8C,D). The completion of wall development in the inter-aperture regions was marked by the return of reinforcing polymers. The return of cellulose to these regions was a relatively late event, evident in the return of clacofluor white signal at the 8 mm stage when exine deposition was already complete. The later stages of pollen development were also associated with changes in pectin content. In particular, the LM19 pollen wall signal was weak in the bicellular pollen stage but much more robust in the mature pollen, forming a continuous layer around the pollen grain. This result suggests considerable enzymatic conversion of meHG to dmeHG between these stages. The defective intine produced by Arabidopsis *pme48* mutants, which are compromised in the reduction of meHG, suggests this conversion is important for ultimate intine formation and function during pollen hydration (Leroux et al., 2015).

Despite similarities in the overall process of pollen wall development, different species produce pollen with markedly different forms. This is particularly apparent for the structure of the exine, which is easily observable and, due to the resistance of sporopollenin to degradation, highly stable in the environment. Pollen also varies in the number, shape, and positioning of its apertures, with some groups lacking them entirely (Furness, 2007). Apertures serve not only as the sites for pollen tube initiation but also facilitate hydration, communication with the stigma, and help accommodate volume changes required during dehydration and rehydration (Matamoro-Vidal et al., 2016). Like many dicots, including Arabidopsis, tomato pollen is tri-aperturate, with exine deposition excluded from the aperture regions (Figure 1B). Our observation of pollinated pistils, using SEM, showed all pollen tubes originating at the aperture site, suggesting that tomato pollen germination is constrained to the aperture sites. However, this is not the case for Arabidopsis and several other species in the Brassicaceae where pollen has been observed to germinate at inter-aperture regions, taking the shortest path to the stigma (Edlund et al., 2016). One possible explanation for this difference between obligate-aperturate (tomato) and omni-aperturate (Arabidopsis) pollen may be differences in inter-aperture region pollen wall stiffness (Furness and Rudall, 2004; Edlund et al., 2016). However, this possible difference in mechanical properties cannot be simply due to differences in exine thickness as Arabidopsis pollen exine is thicker (~1 μm) compared with that of tomato (~0.5 μm) (Dobritsa et al., 2011; Li et al., 2017). In careful examination of the pollen surface, we also noted the presence of small pores (38 ± 9 nm), which were also visible in TEM sections as exine channels (Figure 1C). While the mechanism of their development and the exact chemical nature of the lining of these channels are unknown, they are ubiquitous among land plants, and it is hypothesized that they facilitate hydration in advance of pollen germination (Edlund et al., 2004).

Because pollen wall development is a highly dynamic and complex process, understanding how the pollen wall is constructed requires a temporal understanding of the events of pollen wall remodeling. The work presented here describes the changes of the major cell wall polymers during pollen development in tomato (Figure 9) and will serve as a useful reference point for studies of the perturbation of the pollen wall in this important agricultural species.

## Data Availability Statement

The original contributions presented in the study are included in the article/Supplementary Material, further inquiries can be directed to the corresponding author.

## Author Contributions

CM and SJ designed the study and wrote and edited the manuscript. SJ carried out the experiments. Both authors contributed to the article and approved the submitted version.

## Conflict of Interest

The authors declare that the research was conducted in the absence of any commercial or financial relationships that could be construed as a potential conflict of interest.

## Publisher's Note

All claims expressed in this article are solely those of the authors and do not necessarily represent those of their affiliated organizations, or those of the publisher, the editors and the reviewers. Any product that may be evaluated in this article, or claim that may be made by its manufacturer, is not guaranteed or endorsed by the publisher.

## Supplementary Material

Supplemental Figure 1 Structure of the tomato pollen grain surface. **(A)** Scanning electron micrograph (SEM) of tomato pollen, growing a pollen tube. Arrows mark the apertures (Ap) and pollen tube (Pt). **(B)** SEM of a mature pollen grain. **(C)** Close-up of the box insert from **(B)** showing exine patterning. **(D)** SEM of pollen wall. The arrow marks the Sp, exine spines; and Aq, aquapores. Scale bars 10 μm (**A**, top left), 1 μm (**B**, top right), 1 μm (**C**, bottom left), 100 nm (**D**, bottom right).

Supplemental Figure 2 Structure of tomato anther during pollen development. Micrographs of paraffin-embedded anther sections stained with toluidine blue. **(A)** Sections of 2-mm bud. **(B)** Sections of 4-mm bud. **(C)** Sections of 5-mm bud. **(D)** Sections of 6-mm bud. **(E)** Sections of 8-mm bud. The arrow marks the Pe, petal; An, anther; Se, sepal; Pi, pistil; MMC, microspore mother cell; Loc, locule; Te, tetrad; Tap, tapetum; MS, microspore; BCP, bicellular pollen; scale bars **(A)** 75 μm (left) 50 μm (right) **(B–E)** 200 75 μm (left) 50 μm.

Supplemental Figure 3 No primary antibody control for LM antibody staining. Fluorescent micrographs of anther cross sections (500 nm) in LR white, of 8-mm bud anther, stained with Calcofluor white and an FITC-conjugated secondary antibody only. **(A)** Calcofluor white staining (Left). **(B)** FITC-conjugated secondary only. Scale bars 25 μm.

## References

[B1] Aarts M. G. Hodge R. Kalantidis K. Florack D. Wilson Z. A. Mulligan B. J.. (1997). The Arabidopsis MALE STERILITY 2 protein shares similarity with reductases in elongation/condensation complexes. Plant J. 12, 615–623. 10.1046/j.1365-313X.1997.00615.x 9351246

[B2] Aouali N. Laporte P. Clément C. (2001). Pectin secretion and distribution in the anther during pollen development in Lilium. Planta 213, 71–79. 10.1007/s004250000469 11523658

[B3] Bashir M. E. H. Lui J. H. Palnivelu R. Naclerio R. M. Preuss D. (2013). Pollen lipidomics: lipid profiling exposes a notable diversity in 22 allergenic pollen and potential biomarkers of the allergic immune response. PLoS ONE 8:e57566. 10.1371/journal.pone.0057566 23469025 PMC3585183

[B4] Blackmore S. Wortley A. H. Skvarla J. J. Rowley J. R. (2007). Pollen wall development in flowering plants. New Phytol. 174, 483–498. 10.1111/j.1469-8137.2007.02060.x 17447905

[B5] Bosch M. Hepler P. K. (2005). Pectin methylesterases and pectin dynamics in pollen tubes. Plant Cell 17, 3219–3226. 10.1105/tpc.105.037473 16322606 PMC1315365

[B6] Brukhin V. Hernould M. Gonzalez N. Chevalier C. (2003). Flower development schedule in tomato Lycopersicon esculentum cv. sweet cherry. Sex. Plant Reprod. 15, 311–320. 10.1007/s00497-003-0167-7

[B7] Canales C. Bhatt A. M. Scott R. Dickinson H. (2002). EXS, a putative LRR receptor kinase, regulates male germline cell number and tapetal identity and promotes seed development in Arabidopsis. Curr. Biol. 12, 1718–1727. 10.1016/S0960-9822(02)01151-X 12401166

[B8] Cankar K. Kortstee A. Toonen M. A. J. Wolters-Arts M. Houbein R. Mariani C.. (2014). Pectic arabinan side chains are essential for pollen cell wall integrity during pollen development. Plant Biotechnol. J. 12, 492–502. 10.1111/pbi.12156 24428422

[B9] Cannon M. C. Terneus K. Hall Q. Tan L. Wang Y. Wegenhart B. L.. (2008). Self-assembly of the plant cell wall requires an extensin scaffold. Proc. Natl. Acad. Sci. USA. 105, 2226–2231. 10.1073/pnas.0711980105 18256186 PMC2538902

[B10] Carvalho R. F. Campos M. L. Pino L. E. Crestana S. L. Zsögön A. Lima J. E.. (2011). Convergence of developmental mutants into a single tomato model system: “Micro-Tom” as an effective toolkit for plant development research. Plant Methods 7, 1–14. 10.1186/1746-4811-7-18 21714900 PMC3146949

[B11] Chasan R. (1992). Breaching the callose walI. Plant Cell 4, 745–749. 10.1105/tpc.4.7.745

[B12] Chebli Y. Kaneda M. Zerzour R. Geitmann A. (2012). The cell wall of the Arabidopsis pollen tube-spatial distribution, recycling, and network formation of polysaccharides. Plant Physiol. 160, 1940–1955. 10.1104/pp.112.199729 23037507 PMC3510122

[B13] Choi H. Jin J. Choi S. Hwang J. Kim Y. Suh M. C.. (2011). An ABCG/WBC-type ABC transporter is essential for transport of sporopollenin precursors for exine formation in developing pollen. Plant J. 65, 181–193. 10.1111/j.1365-313X.2010.04412.x 21223384

[B14] Christiaens S. Van Buggenhout S. Ngouémazong E. D. Vandevenne E. Fraeye I. Duvetter T.. (2011). Anti-homogalacturonan antibodies: a way to explore the effect of processing on pectin in fruits and vegetables? Food Res. Int. 44, 225–234. 10.1016/j.foodres.2010.10.031

[B15] Dobritsa A. A. Geanconteri A. Shrestha J. Carlson A. Kooyers N. Coerper D.. (2011). A large-scale genetic screen in Arabidopsis to identify genes involved in pollen exine production. Plant Physiol. 157, 947–970. 10.1104/pp.111.179523 21849515 PMC3192556

[B16] Dobritsa A. A. Reeder S. H. (2017). Formation of pollen apertures in Arabidopsis requires an interplay between male meiosis, development of INP1-decorated plasma membrane domains, and the callose wall. Plant Signal. Behav. 12, 1–4. 10.1080/15592324.2017.1393136 29173018 PMC5792127

[B17] Dong X. Hong Z. Sivaramakrishnan M. Mahfouz M. Verma D. P. S. (2005). Callose synthase (CalS5) is required for exine formation during microgametogenesis and for pollen viability in Arabidopsis. Plant J. 42, 315–328. 10.1111/j.1365-313X.2005.02379.x 15842618

[B18] Edlund A. F. Swanson R. Preuss D. (2004). Pollen and stigma structure and function: The role of diversity in pollination. Plant Cell 16, S84–S97. 10.1105/tpc.015800 15075396 PMC2643401

[B19] Edlund A. F. Zheng Q. Lowe N. Kuseryk S. Ainsworth K. L. Lyles R. H.. (2016). Pollen from Arabidopsis thaliana and other Brassicaceae are functionally omniaperturate. Am. J. Bot. 103, 1006–1019. 10.3732/ajb.1600031 27335390

[B20] El-Ghazaly G. Jensen W. A. (1986). Studies of the development of wheat (*Triticum aestivum*) pollen:I. formation of the pollen wall and ubisch bodies. Grana 25, 1–29. 10.1080/00173138609429929

[B21] Fang K. Wang Y. Yu T. Zhang L. Baluška F. Šamaj J.. (2008). Isolation of de-exined pollen and cytological studies of the pollen intines of *Pinus bungeana* Zucc. Ex Endl. and Picea wilsonii Mast. Flora: Morphol. Distrib. Funct. Ecol. Plants 203, 332–340. 10.1016/j.flora.2007.04.007

[B22] Flores-Tornero M. Anoman A. D. Ros R. (2015). Lack of phosphoserine phosphatase activity alters pollen and tapetum development in *Arabidopsis thaliana*. Plant Sci. 235, 81–88. 10.1016/j.plantsci.2015.03.001 25900568

[B23] Francis K. E. Lam S. Y. Copenhaver G. P. (2006). Separation of arabidopsis pollen tetrads is regulated by *QUARTET1*, a pectin methylesterase gene. Plant Phisiol. 142, 1004–1013. 10.1104/pp.106.085274 16980565 PMC1630721

[B24] Furness C. A. (2007). Why does some pollen lack apertures? A review of inaperturate pollen in eudicots. Bot. J. Linnean Soc. 155, 29–48. 10.1111/j.1095-8339.2007.00694.x

[B25] Furness C. A. Rudall P. J. (2004). Pollen aperture evolution - A crucial factor for eudicot success? Trends Plant Sci. 9, 154–158. 10.1016/j.tplants.2004.01.001 15003239

[B26] Giorno F. Wolters-Arts M. Grillo S. Scharf K. D. Vriezen W. H. Mariani C. (2010). Developmental and heat stress-regulated expression of HsfA2 and small heat shock proteins in tomato anthers. J. Exp. Bot. 61, 453–462. 10.1093/jxb/erp316 19854799 PMC2803211

[B27] Giorno F. Wolters-Arts M. Mariani C. Rieu I. (2013). Ensuring reproduction at high temperatures: the heat stress response during anther and pollen development. Plants 2, 489–506. 10.3390/plants2030489 27137389 PMC4844380

[B28] Gómez J. F. Talle B. Wilson Z. A. (2015). Anther and pollen development: A conserved developmental pathway. J. Integr. Plant Biol. 57, 876–891. 10.1111/jipb.12425 26310290 PMC4794635

[B29] Herburger K. Holzinger A. (2016). Aniline blue and calcofluor white staining of callose and cellulose in the streptophyte green algae Zygnema and Klebsormidium. Bio-Protocol 6, 6–10. 10.21769/BioProtoc.1969 27785458 PMC5076763

[B30] Heslop-Harrison J. (1968). Pollen wall development. Science 161, 230–237. 10.1126/science.161.3838.230 5657325

[B31] Hess M. W. (1993). Cell-wall development in freeze-fixed pollen: Intine formation of *Ledebouria socialis* (Hyacinthaceae). Planta 189, 139–149. 10.1007/BF00201354

[B32] Hess M. W. Frosch A. (1994). Subunits of forming pollen exine and Ubisch bodies as seen in freeze substituted *Ledebouria socialis* Roth (Hyacinthaceae). Protoplasma 182, 10–14. 10.1007/BF01403683

[B33] Hsiao A. Yeung E. C. Ye Z. Chye M. (2015). The Arabidopsis Cytosolic Acyl-CoA-Binding proteins play combinatory roles in pollen development. Plant Cell Physiol. 56, 322–333. 10.1093/pcp/pcu163 25395473

[B34] Ishiguro S. Nishimori Y. Yamada M. Saito H. Suzuki T. Nakagawa T.. (2010). The Arabidopsis *FLAKY POLLEN1* gene encodes a 3-hydroxy-3-methylglutaryl- coenzyme a synthase required for development of tapetum-specific organelles and fertility of pollen grains. Plant Cell Physiol. 51, 896–911. 10.1093/pcp/pcq068 20484369

[B35] Jacobowitz J. R. Doyle W. C. Weng J. (2019). PRX9 and PRX40 Are extensin peroxidases essential for maintaining tapetum and microspore cell wall integrity during arabidopsis anther development. Plant Cell 31, 848–861. 10.1105/tpc.18.00907 30886127 PMC6501601

[B36] Jiang J. Zhang Z. Cao J. (2013). Pollen wall development: the associated enzymes and metabolic pathways. Plant Biol. 15, 249–263. 10.1111/j.1438-8677.2012.00706.x 23252839

[B37] Kravchik M. Stav R. Belausov E. Arazi T. (2019). Functional characterization of microRNA171 family in Tomato. Plants 8:10. 10.3390/plants8010010 30621201 PMC6358981

[B38] Lee J. T. Y. Chow K. L. (2012). SEM sample preparation for cells on 3D scaffolds by freeze-drying and HMDS. Scanning 34, 12–25. 10.1002/sca.20271 22532079

[B39] Leroux C. Bouton S. Fabrice T. N. Mareck A. Guénin S. Fournet F.. (2015). PECTIN METHYLESTERASE48 is involved in Arabidopsis pollen grain germination. Plant Physiol. 167, 367–380. 10.1104/pp.114.250928 25524442 PMC4326738

[B40] Li W. L. Liu Y. Douglas C. J. (2017). Role of glycosyltransferases in pollen wall primexine formation and exine patterning. Plant Physiol. 173, 167–182. 10.1104/pp.16.00471 27495941 PMC5210704

[B41] Luis da Costa M. Lopes A. L. Amorim M. I. Coimbra S. (2017). Immunolocalization of AGPs and pectins in *Quercus suber*gamethophytic structures. Plant Germline Develop. Methods Protoc. Methods Mol. Bio. 1669, 117–137. 10.1007/978-1-4939-7286-9_11 28936655

[B42] Lyu M. Yu Y. Jiang J. Song L. Liang Y. Ma Z.. (2015). *BcMF26a* and *BcMF26b*are duplicated polygalacturonase genes with divergent expression patterns and functions in pollen development and pollen tube formation in *Brassica campestris*. PLoS ONE 10:e0131173. 10.1371/journal.pone.0131173 26153985 PMC4495986

[B43] Ma X. Wu Y. Zhang G. (2021). Formation pattern and regulatory mechanisms of pollen wall in Arabidopsis. J. Plant Physiol. 260, 153388. 10.1016/j.jplph.2021.153388 33706055

[B44] Matamoro-Vidal A. Raquin C. Brisset F. Colas H. Izac B. Albert B.. (2016). Links between morphology and function of the pollen wall: an experimental approach. Bot. J. Linnean Soc. 180, 478–490. 10.1111/boj.12378

[B45] Mollet J. C. Leroux C. Dardelle F. Lehner A. (2013). Cell wall composition, biosynthesis and remodeling during pollen tube growth. Plants Basel 2, 107–147. 10.3390/plants2010107 27137369 PMC4844286

[B46] Müller F. Xu J. Kristensen L. Wolters-Arts M. De Groot P. F. M. Jansma S. Y.. (2016). High-temperature-induced defects in tomato (*Solanum lycopersicum*) anther and pollen development are associated with reduced expression of B-class floral patterning genes. PLoS ONE 11:e0167614. 10.1371/journal.pone.0167614 27936079 PMC5147909

[B47] Ogawa M. Kay P. Wilson S. Swain S. M. (2009). ARABIDOPSIS DEHISCENCE ZONE POLYGALACTURONASE1 required for cell separation during reproductive development in Arabidopsis. Plant Cell 21, 216–233. 10.1105/tpc.108.063768 19168715 PMC2648098

[B48] Owen H. A. Makaroff C. A. (1995). Ultrastructure of microsporogenesis and microgametogenesis in *Arabidopsis thaliana* (L.) Heynh. ecotype Wassilewskija (Brassicaceae). Protoplasma 185, 7–21. 10.1007/BF01272749

[B49] Paxson-sowders D. M. Dodrill C. H. Owen H. A. Makaroff C. A. (2001). DEX1, a novel plant protein, is required for exine pattern formation during pollen development in Arabidopsis. Plant Physiol. 127, 1739–1749. 10.1104/pp.010517 11743117 PMC133577

[B50] Paxson-Sowders D. M. Owen H. A. Makarow C. A. (1997). A comparative ultrastructural analysis of exine pattern development in wild-type Arabidopsis and a mutant defective in pattern formation. Protoplasma 198, 53–65. 10.1007/BF01282131

[B51] Persson S. Paredez A. Carroll A. Palsdottir H. Doblin M. Poindexter P.. (2007). Genetic evidence for three unique components in primary cell-wall cellulose synthase complexes in Arabidopsis. Proc. Natl. Acad. Sci. U.S.A. 104, 15566–15571. 10.1073/pnas.0706592104 17878302 PMC2000526

[B52] Petersen B. L. Macalister C. A. Petersen B. L. Ulvskov P. (2021). Plant protein O-arabinosylation. Front. Plant Sci. 12:645219. 10.3389/fpls.2021.645219 33815452 PMC8012813

[B53] Phan H. A. Iacuone S. Li S. F. Parish R. W. (2011). The MYB80 transcription factor is required for pollen development and the regulation of tapetal programmed cell death in *Arabidopsis thaliana*. Plant Cell 23, 2209–2224. 10.1105/tpc.110.082651 21673079 PMC3160043

[B54] Picken A. J. F. (1984). A review of pollination and fruit set in the tomato (*Lycopersicon esculentum Mill*.). J. Hortic. Sci. 59, 1–13. 10.1080/00221589.1984.11515163

[B55] Piffanelli P. Murphy D. J. (1998). Novel organelles and targeting mechanisms in the anther tapetum. Trends Plant Sci. 3, 250–252. 10.1016/S1360-1385(98)01260-6

[B56] Piffanelli P. Ross J. H. E. Murphy D. J. (1998). Biogenesis and function of the lipidic structures of pollen grains. Sex. Plant Reprod. 11, 65–80. 10.1007/s004970050122

[B57] Polowick P. L. Sawhney V. K. (1992). Ultrastructural changes in the cell wall, nucleus and cytoplasm of pollen mother cells during meiotic prophase I in *Lycopersicon esculentum* (Mill.). Protoplasma 169, 139–147. 10.1007/BF01323613

[B58] Polowick P. L. Sawhney V. K. (1993a). An ultrastructural study of pollen development in tomato (*Lycopersicon esculentum*). *I*. Tetrad to early binucleate microspore stage. Can. J. Bot. 71, 1039–1047. 10.1139/b93-120

[B59] Polowick P. L. Sawhney V. K. (1993b). Differentiation of the tapetum during microsporogenesis in tomato (*Lycopersicon esculentum Mill*.), with special reference to the tapetal cell wall. Ann. Bot. 72, 595–605. 10.1006/anbo.1993.1150

[B60] Pressman E. Peet M. M. Pharr D. M. (2002). The effect of heat stress on tomato pollen characteristics is associated with changes in carbohydrate concentration in the developing anthers. Ann. Bot. 90, 631–636. 10.1093/aob/mcf240 12466104 PMC4240456

[B61] Preuss D. Lemieux B. Yen G. Davis R. W. (1993). A conditional sterile mutation eliminates surface components from Arabidopsis pollen and disrupts cell signaling during fertilization. Genes Develop. 7, 974–985. 10.1101/gad.7.6.974 8504936

[B62] Quilichini T. D. Douglas C. J. Samuels A. L. (2014). New views of tapetum ultrastructure and pollen exine development in *Arabidopsis thaliana*. Ann. Bot. 114, 1189–1201. 10.1093/aob/mcu042 24723448 PMC4195548

[B63] Quilichini T. D. Friedmann M. C. Samuels A. L. Douglas C. J. (2010). ATP-Binding Cassette Transporter G26 is required for male fertility and pollen exine formation in Arabidopsis. Plant Physiol. 154, 678–690. 10.1104/pp.110.161968 20732973 PMC2949020

[B64] Rejón J. D. Delalande F. Schaeffer-Reiss C. Alch,é J. de D. Rodríguez-García M. I.. (2016). The pollen coat proteome: at the cutting edge of plant reproduction. Proteomes 4, 1–23. 10.3390/proteomes4010005 28248215 PMC5217362

[B65] Renzaglia K. S. Lopez R. A. Welsh R. D. Owen H. A. Merced A. (2020). Callose in sporogenesis: novel composition of the inner spore wall in hornworts. Plant System. Evol. 306, 1–9. 10.1007/s00606-020-01631-5 34079158 PMC8167838

[B66] Rhee S. Y. Osborne E. Poindexter P. D. Somerville C. R. (2003). Microspore separation in the *quartet3* mutants of Arabidopsis is impaired by a defect in a developmentally regulated polygalacturonase required for pollen mother cell wall degradation. Plant Physiol. 133, 1170–1180. 10.1104/pp.103.028266 14551328 PMC281612

[B67] Schnabelrauch L. S. Kieliszewski M. J. Upham B. L. Alizedeh H. Lamport D. T. A. (1996). Isolation of pl4.6 extensin peroxidase from tomato cell suspension cultures and identification of Val-Tyr-Lys as putative intramolecular cross-link site. Plant J. 9, 477–489. 10.1046/j.1365-313X.1996.09040477.x 8624511

[B68] Scott R. J. Spielman M. Dickinson H. G. (2004). Stamen structure and function. Plant Cell 16, 46–61. 10.1105/tpc.017012 PMC2643399 15131249

[B69] Shi J. Cui M. Yang L. Kim Y. J. Zhang D. (2015a). Genetic and biochemical mechanisms of pollen wall development. Trends Plant Sci. 20, 741–753. 10.1016/j.tplants.2015.07.010 26442683

[B70] Shi J. Tan H. Yu X. Liu Y. Liang W. Ranathunge K. Franke R. B.. (2011). Defective pollen wall is required for anther and microspore development in rice and encodes a fatty acyl carrier protein reductase. Plant Cell 23, 2225–2246. 10.1105/tpc.111.087528 21705642 PMC3160036

[B71] Shi X. Sun X. Zhang Z. Feng D. Zhang Q. Han L.. (2015b). GLUCAN SYNTHASE-LIKE 5 (GSL5) plays an essential role in male fertility by regulating callose metabolism during microsporogenesis in rice. Plant Cell Physiol. 5, 497–509. 10.1093/pcp/pcu193 25520407

[B72] Souza C. D. A. Kim S. S. Koch S. Kienow L. Schneider K. Mckim S. M.. (2009). A novel Fatty Acyl-CoA synthetase is required for pollen development and sporopollenin biosynthesis in Arabidopsis. Plant Cell 21, 507–525. 10.1105/tpc.108.062513 19218397 PMC2660628

[B73] Sridharan G. Shankar A. A. (2012). Toluidine blue : A review of its chemistry and clinical utility. J. Oral Maxillofacial Pathol. 16, 251–255. 10.4103/0973-029X.99081 22923899 PMC3424943

[B74] Suárez-Cervera M. Arcalís E. Le Thomas A. Seoane-Camba J. A. (2002). Pectin distribution pattern in the apertural intine of *Euphorbia peplus L. (Euphorbiaceae)* pollen. Sex. Plant Reprod. 14, 291–298. 10.1007/s00497-001-0121-5

[B75] Suzuki T. Narciso J. O. Zeng W. van de Meene A. Yasutomi M. Takemura S.. (2017). Kns4/upex1: a type II arabinogalactan β-(1,3)-galactosyltransferase required for pollen exine development. Plant Physiol. 173, 183–205. 10.1104/pp.16.01385 27837085 PMC5210738

[B76] Takebe N. Nakamura A. Watanabe T. Miyashita A. Satoh S. Iwai H. (2020). Cell wall Glycine-rich Protein2 is involved in tapetal differentiation and pollen maturation. J. Plant Res. 133, 883–895. 10.1007/s10265-020-01223-x 32929552

[B77] Taylor L. P. Hepler P. K. (1997). Pollen germination and tube growth. Ann. Rev. Plant Physiol. Plant Molec. Bio. 48, 461–491. 10.1146/annurev.arplant.48.1.461 15012271

[B78] Verhertbruggen Y. Marcus S. E. Haeger A. Ordaz-ortiz J. J. Knox J. P. (2009). An extended set of monoclonal antibodies to pectic homogalacturonan. Carbohydr. Res. 344, 1858–1862. 10.1016/j.carres.2008.11.010 19144326

[B79] Verlag F. Wakabayashi K. Hoson T. Huber D. J. (2003). Methyl de-esterification as a major factor regulating the extent of pectin depolymerization during fruit ripening: a comparison of the action of avocado (*Persea americana*) and tomato (*Lycopersicon esculentum*) polygalacturonases. J. Plant Physiol. 673, 667–673. 10.1078/0176-1617-00951 12872489

[B80] Wan L. Zha W. Cheng X. Liu C. Lv L. Liu C.. (2011). A rice beta-1,3-glucanase gene Osg1 is required for callose degradation in pollen development. Planta 309–323. 10.1007/s00425-010-1301-z 21046148

[B81] Wang R. Dobritsa A. A. (2018). Exine and Aperture patterns on the pollen surface: their formation and roles in plant reproduction. Ann. Plant Rev. 1, 1–40. 10.1002/9781119312994.apr0625

[B82] Yu J. Meng Z. Liang W. Behera S. Kudla J. Tucker M. R.. (2016). A Rice Ca 2+ Binding protein is required for tapetum function and pollen formation. Plant Physiol. 172, 1772–1786. 10.1104/pp.16.01261 27663411 PMC5100779

[B83] Zhang D. Chen W. Yu X. Zhang K. Shi J. Oliveira S.. (2011). *Male Sterile2* encodes a plastid-localized fatty acyl carrier protein reductase required for pollen exine development in Arabidopsis. Plant Phisiol. 157, 842–853. 10.1104/pp.111.181693 21813653 PMC3192575

[B84] Zhang X. Zhao G. Tan Q. Yuan H. Betts N. Zhu L.. (2020). Rice pollen aperture formation is regulated by the interplay between OsINP1 and OsDAF1. Nat. Plants 6, 394–403. 10.1038/s41477-020-0630-6 32284546

[B85] Zinkl G. M. Zwiebel B. I. Grier D. G. Preuss D. (1999). Pollen-stigma adhesion in Arabidopsis : a species-specific interaction mediated by lipophilic molecules in the pollen exine. Development 5440, 5431–5440. 10.1242/dev.126.23.5431 10556067

