# Supplementary figures and images for: Sequential Deposition and Remodeling of Cell Wall Polymers During Tomato Pollen Development
Source: Front Plant Sci. 2021 Jul 27;12:703713. doi: 10.3389/fpls.2021.703713 (PMC8354551; doi:10.3389/fpls.2021.703713)

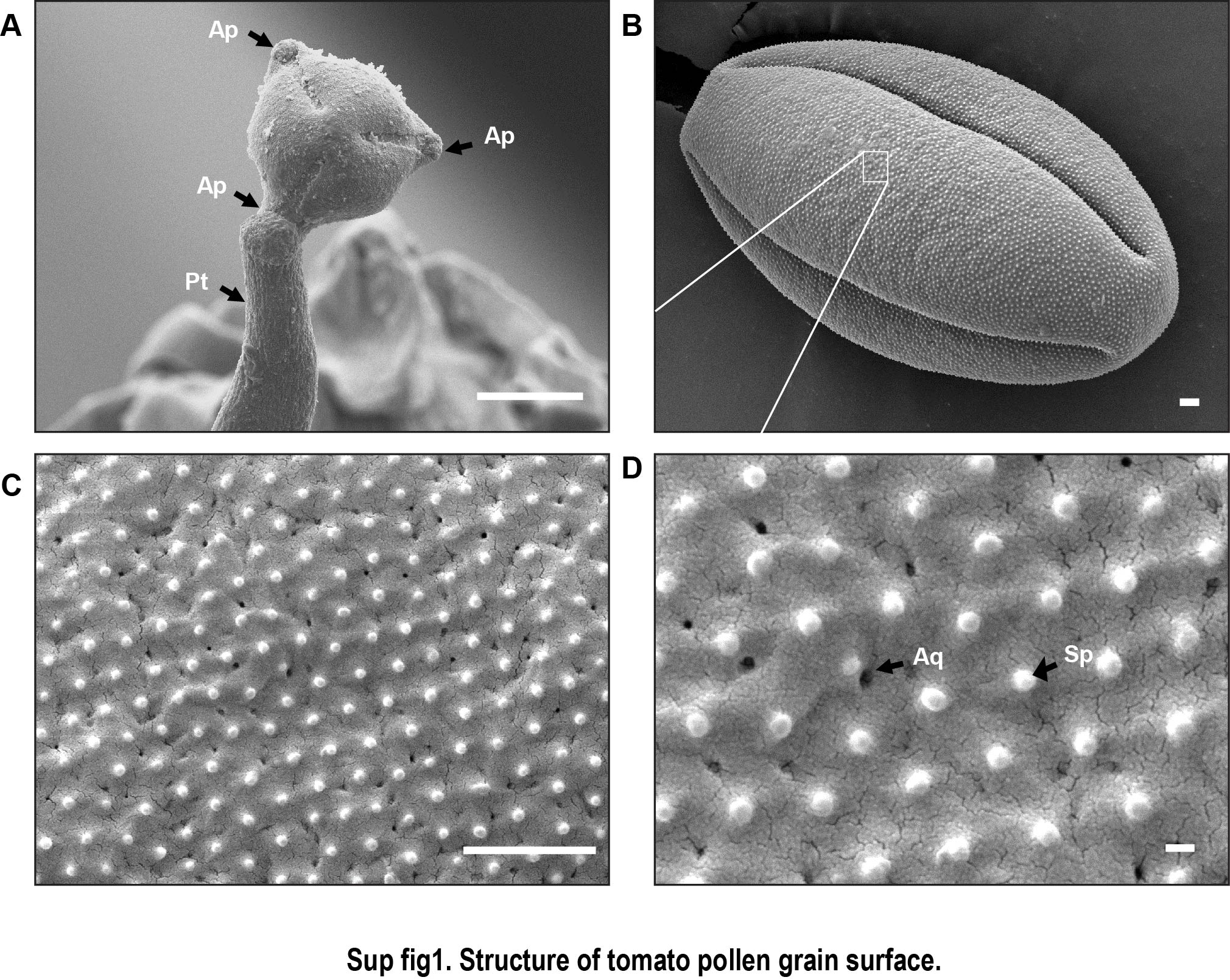

Supplement: Supplemental Figure 1 — Structure of the tomato pollen grain surface. (A) Scanning electron micrograph (SEM) of tomato pollen, growing a pollen tube. Arrows mark the apertures (Ap) and pollen tube (Pt). (B) SEM of a mature pollen grain. (C) Close-up of the box insert from (B) showing exine patterning. (D) SEM of pollen wall. The arrow marks the Sp, exine spines; and Aq, aquapores. Scale bars 10 μm (A, top left), 1 μm (B, top right), 1 μm (C, bottom left), 100 nm (D, bottom right). [file Image_1.JPEG]

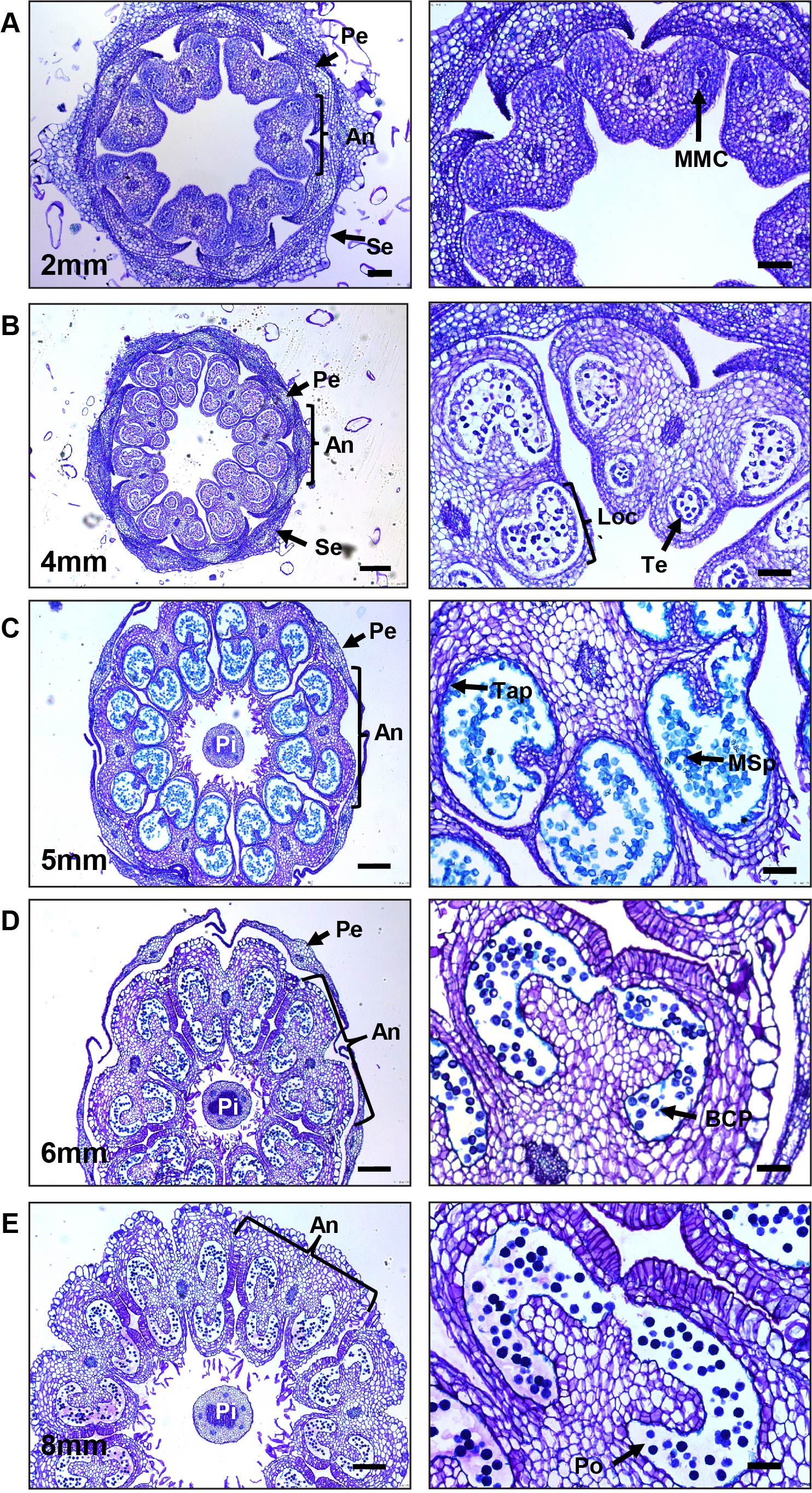

Supplement: Supplemental Figure 2 — Structure of tomato anther during pollen development. Micrographs of paraffin-embedded anther sections stained with toluidine blue. (A) Sections of 2-mm bud. (B) Sections of 4-mm bud. (C) Sections of 5-mm bud. (D) Sections of 6-mm bud. (E) Sections of 8-mm bud. The arrow marks the Pe, petal; An, anther; Se, sepal; Pi, pistil; MMC, microspore mother cell; Loc, locule; Te, tetrad; Tap, tapetum; MS, microspore; BCP, bicellular pollen; scale bars (A) 75 μm (left) 50 μm (right) (B–E) 200 75 μm (left) 50 μm. [file Image_2.JPEG]

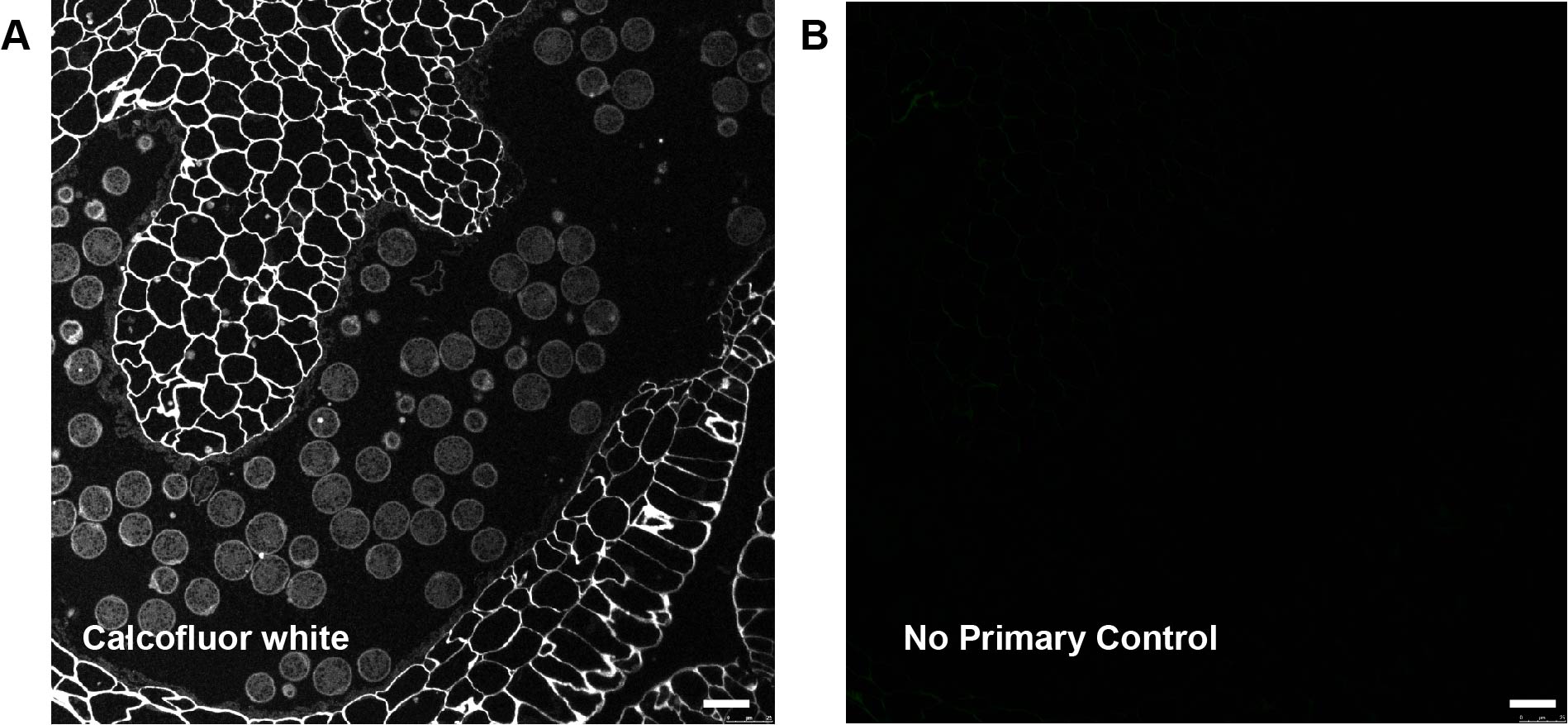

Supplement: Supplemental Figure 3 — No primary antibody control for LM antibody staining. Fluorescent micrographs of anther cross sections (500 nm) in LR white, of 8-mm bud anther, stained with Calcofluor white and an FITC-conjugated secondary antibody only. (A) Calcofluor white staining (Left). (B) FITC-conjugated secondary only. Scale bars 25 μm. [file Image_3.JPEG]
